# Acute-Phase Neurofilament Light and Glial Fibrillary Acidic Proteins in Cerebrospinal Fluid Predict Long-Term Outcome After Severe Traumatic Brain Injury

**DOI:** 10.1007/s12028-024-01998-0

**Published:** 2024-05-20

**Authors:** Emma Andersson, Martin Öst, Keti Dalla, Henrik Zetterberg, Kaj Blennow, Bengt Nellgård

**Affiliations:** 1 Department of Anesthesiology and Intensive Care Medicine, Institution of Clinical Sciences, Gothenburg University, Gothenburg, Sweden; 2 Department of Psychiatry and Neurochemistry, Institute of Neuroscience and Physiology, The Sahlgrenska Academy at the University of Gothenburg, Mölndal, Sweden; 3 Clinical Neurochemistry Laboratory, Sahlgrenska University Hospital, Mölndal, Sweden; 4 Department of Neurodegenerative Disease, UCL Institute of Neurology, Queen Square, London, UK; 5 UK Dementia Research Institute at UCL, London, UK; 6 Hongkong Center for Neurodegenerative Diseases, Science Park, Hongkong China

**Keywords:** Glial fibrillary acidic protein, Intermediate filaments, Brain injuries, Traumatic, Neurofilament proteins, Glasgow Outcome Scale

## Abstract

**Background:**

This study investigated trajectory profiles and the association of concentrations of the biomarkers neurofilament light (NfL) and glial fibrillary acidic protein (GFAP) in ventricular cerebrospinal fluid (CSF) with clinical outcome at 1 year and 10–15 years after a severe traumatic brain injury (sTBI).

**Methods:**

This study included patients with sTBI at the Neurointensive Care Unit at Sahlgrenska University Hospital, Gothenburg, Sweden. The injury was regarded as severe if patients had a Glasgow Coma Scale ≤ 8 corresponding to Reaction Level Scale ≥ 4. CSF was collected from a ventricular catheter during a 2-week period. Concentrations of NfL and GFAP in CSF were analyzed with enzyme-linked immunosorbent assay. The Glasgow Outcome Scale (GOS) was used to assess the 1-year and 10–15-year outcomes. After adjustment for age and previous neurological diseases, logistic regression was performed for the outcomes GOS 1 (dead) or GOS 2–5 (alive) and GOS 1–3 (poor) or GOS 4–5 (good) versus the independent continuous variables (NfL and GFAP).

**Results:**

Fifty-three patients with sTBI were investigated; forty-seven adults are presented in the article, and six children (aged 7–18 years) are described in Supplement 1. The CSF concentrations of NfL gradually increased over 2 weeks post trauma, whereas GFAP concentrations peaked on days 3–4. Increasing NfL and GFAP CSF concentrations increased the odds of GOS 1–3 outcome 1 year after trauma (odds ratio [OR] 1.73, 95% confidence interval [CI] 1.07–2.80, *p* = 0.025; and OR 1.61, 95% CI 1.09–2.37, *p* = 0.016, respectively). Similarly, increasing CSF concentrations of NfL and GFAP increased the odds for GOS 1–3 outcome 10–15 years after trauma (OR 2.04, 95% CI 1.05–3.96, *p* = 0.035; and OR 1.60, 95% CI 1.02–2.00, *p* = 0.040).

**Conclusions:**

This study shows that initial high concentrations of NfL and GFAP in CSF are both associated with higher odds for GOS 1–3 outcome 1 year and 10–15 years after an sTBI, implicating its potential usage as a prognostic marker in the future.

**Supplementary Information:**

The online version contains supplementary material available at 10.1007/s12028-024-01998-0.

## Introduction

Severe traumatic brain injury (sTBI) (i.e., Glasgow Coma Scale [GCS] of ≤ 8) is a condition that includes focal contusions, intradural and extradural hematomas, and diffuse axonal injuries [1]. These injuries often lead to severe lifelong neurological deficits or even death. sTBI is associated with changes in the levels of biomarkers in cerebrospinal fluid (CSF), indicating different pathology and brain cell origin [2, 3].

In neurons, neurofilaments, including neurofilament light (NfL), are found in axons and are involved in axonal transport, axonal growth, and cytoskeleton plasticity [3, 4]. When neurons are exposed to trauma, intracellular damage leads to axonal swelling and disconnection and the NfL passes into the CSF [5]. Glial fibrillary acidic protein (GFAP) is expressed by astrocytes, and when injured, GFAP is released into the CSF [2]. Further, as a response to trauma, GFAP synthesis increases, contributing to the extension and thickening of the astrocytic processes observed around damaged areas. Astrocytes provide both functional and structural support to neurons and the blood–brain barrier (BBB) [2].

Previously, parts of this sTBI population have been investigated to relate GFAP, S100b, and NfL in serum as well as β-amyloid, amyloid precursor protein, and tau in CSF and the gene APOE ε4 with 1-year outcome [6–11]. CSF NfL was analyzed by Shahim et al. [6] and compared with serum NfL levels but was never related to outcome.

In the present study, we analyzed NfL, a biomarker for of axonal injury, and GFAP, a biomarker of astrocyte injury and response, in ventricular CSF [2, 3]. We explored the trajectory profiles of NfL and GFAP in the CSF during the initial 2 weeks in patients with sTBI. Further, our hypothesis was that concentrations of NfL and GFAP in the CSF analyzed early after an sTBI were able to predict 1-year and 10–15-year patient outcome.

## Methods

### Patients

The study was performed in accordance with the provisions of the Helsinki Declaration. The University Hospital Medical Ethics Committee, Gothenburg, Sweden, approved the initial study protocol (S-161 00) as well as a supplementary application for the secondary assessment (438-15). The investigation was registered on ClinicalTrials.gov (identifier NCT05138692). Informed written consent was obtained from each patient or next of kin.

Patients with an sTBI were included between October 2000 and November 2004. All the included patients had been admitted to the neurointensive care unit (NICU) at Sahlgrenska University Hospital within 48 h after the insult. The injury was regarded as severe (sTBI) if patients were unconscious, the GCS [12] was ≤ 8, or the Reaction Level Scale [13] was ≥ 4, corresponding to a GCS ≤ 8. In addition, the following inclusion criteria had to be fulfilled: (1) need for artificial ventilation, (2) need for insertion of a ventricular catheter for intracranial pressure (ICP) monitoring, and (3) residing in Sweden for follow-up. Finally, both children (> 7 years) and adults were included. The pediatric population is presented separately in Supplement 1.

After a clinical and radiologic evaluation using computed tomography (CT), patients received a ventricular catheter for ICP monitoring and, if needed, therapeutic CSF drainage. Patients were treated according to a standardized protocol, the Lund concept [14]. The Lund concept is a therapy with the main goal to decrease ICP and improve microcirculation. It is ICP driven, not cerebral perfusion pressure (CPP) driven, with a goal of having an ICP of < 20 mm Hg (27cmH_2_O), concomitant with a CPP > 50 mm Hg. This is achieved by keeping colloid pressure high (albumin) combined with normalized electrolytes, diuretics, heavy sedation, temperature regulation, and body positioning [14]. If ICP and CPP could not be kept within decided limits and/or an eminent herniation was at hand, hematomas were surgically evacuated and/or decompressive hemicraniectomy was performed. Physiologic and laboratory variables were continuously monitored and adjusted to be kept within predefined limits, described in previously published investigations [6–11, 15].

### Assessments

CSF samples were collected intermittently on days 0 (trauma day), 1–4, 6, and 8 and once during days 11–18. Because of the risk of herniation, some CSF samples were not obtained because of high ICP, decided by the neurosurgeon in charge. CSF samples were aliquoted and then frozen at − 70 °C until analyzed. Concentrations of NfL and GFAP in the CSF were measured using enzyme-linked immunosorbent assays (ELISAs), Uman Diagnostics ELISA for NfL and Rosengrens in-house ELISA for GFAP [16, 17]. Within-run and between-run variability have a coefficient of variability (CV)  < 10%. The analyses were performed in 2006.

Data on previous diseases, type of injury, operations, and rehab were mainly collected from medical records. The Marshall classification was used to categorize the damage seen on initial CT scans and was assessed by neuroradiologists [18].

A neurologist blinded to biomarker concentrations performed the first clinical assessment at 1 year post injury (2001–2006). The outcome was assessed using the extended eight-graded version of the Glasgow Outcome Scale (GOS) and was then converted to the five-graded version: GOS 1 = dead, GOS 2 = vegetative state, GOS 3 = severe disability, GOS 4 = moderate disability, and GOS 5 = good recovery. The GOS was dichotomized into GOS 1–3 (poor) or GOS 4–5 (good) outcome and then further into GOS 1 (dead) or GOS 2–5 (alive) [19, 20]. King’s Outcome Scale for Childhood Head Injury was used to assess outcome in children 1 year after trauma [21].

For the second assessment, 10–15 years post trauma, patients were checked against the Swedish Tax Agency and medical records to retrieve information about whether they were still alive. In 2015, through telephone-based interviews, one investigator, who was different from the first interviewer, conducted the second assessment according to the GOS protocol [19, 20]. If patients were unable to provide sufficient information about their GOS, this information was instead collected from relatives or caregivers.

### Statistics

The distributions of skewed continuous variables, such as NfL and GFAP, are given as the median, first quartile, and third quartile in tables and with the addition of minimum and maximum in boxplots. Unadjusted correlations were analyzed with Spearman’s rank correlation (*r*_s_), and adjusted correlations were analyzed with Spearman’s partial correlation coefficient. For comparisons of NfL and GFAP between two groups, Fisher’s permutation test was used for unadjusted analyses, and analysis of covariance was used for adjusted analyses, both on log-transformed values to describe the difference between two groups regarding the NfL and GFAP fold change, and the 95% confidence interval (CI) is given, calculated as the antilogarithm of the mean difference with the 95% CI on the log scale. Fold change should be interpreted as the number of times greater or less by which one group is compared with the other. The Mann–Whitney *U*-test was used to compare the concentration differences in continuous variables (NfL and GFAP) per sample day between GOS 1–3 (poor) and GOS 3–5 (good) outcome and GOS 1 (dead) and GOS 2–5 (alive). The analyses of correlations between NfL and GFAP against the GOS and the comparison between two GOS groups regarding NfL and GFAP were adjusted for confounders. Confounders are the baseline variables that correlated significantly (Spearman correlation test) with both the GOS and the analyzed NfL and/or GFAP values, on the condition that the variable can affect NfL and/or GFAP concentrations in CSF and GOS results. Adjusted logistic regression was based on the dependent variables of GOS 1 or GOS 2–5 and GOS 1–3 or GOS 4–5 outcome versus the independent continuous variables (NfL and GFAP) on the log scale presented as the odds ratio (OR) with the 95% CI, *p* values, and the area under the receiver operating characteristic curve. The cutoff values and Youden index were calculated on the best sum of sensitivity and specificity. The number of deaths (*n* = 9) 1 year after trauma was too small to allow for adjustment analysis. When appropriate, 1-year outcome analyses were adjusted for age and 10–15-year outcome analyses were adjusted for age and pretrauma neurological diseases. All analyses of NfL and GFAP were made on the highest concentration of each patient. All significance tests were two-sided and conducted at the 5% significance level. All statistical analyses were performed using SAS system version 9.4.

## Results

### Patient Characteristics

A total of 124 eligible patients were available for inclusion, whereas 3 patients were excluded because of the lack of a ventricular catheter, 6 patients or their next of kin failed to give informed consent, 1 patient did not reside in Sweden, and 12 patients, after reconsideration, did not fulfill the criteria for an sTBI. Additionally, three patients were excluded at the 10–15-year follow-up because the 1-year data were missing in two patients and one did not reside in Sweden. Further, of the remaining 99 patients, 46 were excluded because of the lack of CSF samples for biomarker analysis. As a result, 53 patients were included. Six patients were children at the time of injury, and this pediatric population is described separately in Supplement 1. The remaining 47 adult patients are presented as follows. They had a mean age on admission of 45.4 years (SD 16.1 years), and 83% were men (Table 1). Results including both the pediatric and adult population are presented in corresponding tables and figures in Supplement 2.

**Table 1:** Patient characteristics

		1 year after trauma		10–15 years after trauma	
Variable	Total	GOS 1–3 (poor outcome)	GOS 4–5 (good outcome)	GOS 1–3 (poor outcome)	GOS 4–5 (good outcome)
Included patients, *n* (%)	47 (100.0%)	25 (100.0%)	22 (100.0%)	23 (100.0%)	21 (100.0%)
Glasgow Outcome Scale (GOS), *n* (%)					
GOS 1 (Dead)		9 (36.0%)		18 (78.3%)	
GOS 2 (Vegetative state)		0 (0.0%)		0 (0.0%)	
GOS 3 (Severe disability)		16 (64.0%)		5 (21.7%)	
GOS 4 (Moderate disability)			16 (72.7%)		15 (71.4%)
GOS 5 (Good recovery)			6 (27.3%)		6 (28.6%)
Age at time of trauma					
Mean (SD)	45.4 (16.1)	49.1 (15.6)	41.2 (16.0)	53.1 (14.6)	37.5 (14.8)
Median (min–max)	44.1 (19.6; 76.7)	48.9 (20.8; 76.7)	40 (19.6; 71.8)	56.5 (20.8; 76.7)	36.6 (19.6; 71.8)
Sex, *n* (%)					
Male	39 (83.0%)	19 (76.0%)	20 (90.9%)	17 (73.9%)	19 (90.5%)
Female	8 (17.0%)	6 (24.0%)	2 (9.1%)	6 (26.1%)	2 (9.5%)
Pre-trauma diseases, *n* (%)					
Neurological disease, missing = 2	8 (17.8%)	6 (26.1%)	2 (9.1%)	7 (33.3%)	1 (4.8%)
Diabetes mellitus	2 (4.3%)	2 (8.0%)	0 (0.0%)	2 (8.7%)	0 (0.0%)
Heart disease	4 (8.5%)	3 (12.0%)	1 (4.5%)	4 (17.4%)	0 (0.0%)
High blood pressure	3 (6.4%)	2 (8.0%)	1 (4.5%)	2 (8.7%)	1 (4.8%)
Neurosurgery, *n* (%)					
Evacuation of hematomas	21 (44.7%)	13 (52.0%)	8 (36.4.0%)	13 (56.5%)	7 (33.3%)
Evacuation of hematomas and decompressive craniectomy	6 (12.8%)	4 (16.0%)	2 (9.1%)	4 (17.4%)	2 (9.5%)
Decompressive craniectomy	8 (17.0%)	5 (20.0%)	3 (13.6%)	5 (21.7%)	3 (14.3%)
Revision of skull fracture	3 (6.4%)	2 (8.0%)	1 (4.5%)	2 (8.7%)	1 (4.8%)
Dura reconstruction	5 (10.6%)	5 (20.0%)	0 (0.0%)	3 (13.0%)	1 (4.8%)
Type of trauma, *n* (%)					
Isolated head trauma	28 (59.6%)	16 (64.0%)	12 (54.5%)	17 (73.9%)	10 (47.6%)
Multi trauma	19 (40.4%)	9 (36.0%)	10 (45.5%)	6 (26.1%)	11 (52.4%)
Cause of trauma, *n* (%)					
Road traffic accident	23 (48.9%)	11(44.0%)	12 (54.5%)	9 (39.1%)	12 (57.1%)
Fall	15 (31.9%)	8 (32.0%)	7 (31.8%)	9 (39.1%)	5 (23.8%)
Miscellaneous	8 (17.0%)	6 (24.0%)	2 (9.1%)	5 (21.7%)	3 (14.3%)
Assault	1 (2.1%)	0 (0.0%)	1 (4.5%)	0 (0.0%)	1 (4.8%)
Marshall CT-Classification, *n* (%), Missing = 1					
Diffuse injury I (Normal)	0 (0.0%)	0 (0.0%)	0 (0.0%)	0 (0.0%)	0 (0.0%)
Diffuse injury II (Lesions, present cisterns, midline shift 0–5 mm)	13 (28.3%)	6 (24.0%)	7 (33.3%)	6 (26.1%)	7 (35.0%)
Diffuse injury III (Lesions, cisterns compressed, midline shift 0–5 mm)	9 (19.6%)	4 (16.0%)	5 (23.8%)	2 (8.7%)	6 (30.0%)
Diffuse injury IV (Midline shift > 5 mm)	21 (45.7%)	13 (52.0%)	8 (38.1%)	12 (52.2%)	7 (35.0%)
V (Any surgically evacuated lesion)	3 (6.5%)	2 (8.0%)	1 (4.8%)	3 (13.0%)	0 (0.0%)
VI (Non evacuated mass lesion, > 25 cc lesion)	0 (0.0%)	0 (0.0%)	0 (0.0%)	0 (0.0%)	0 (0.0%)
Rehabilitation, *n* (%), Missing = 2	33 (73.3%)	14 (56.0%)	19 (95.0%)	12 (54.5%)	20 (95.2%)

Patient characteristics presented for the whole cohort (total) and in subgroups, GOS 1–3 and GOS 4–5 outcome at both one year and 10–15 years after trauma. At the assessment 10–15 years after trauma, 3 patients did not participate, i.e. 10–15 years after trauma: missing = 3, *n* = 50. Neurological diseases include epilepsy (*n* = 5), transient ischemic attack (*n* = 1) and previous trauma to the head (*n* = 2). For categorical variables *n* (%) is presented. For continuous variables Mean (SD) / Median (Min; Max) is presented. GOS: Glasgow Outcome Scale, *n*: number of observations

Nine patients died during the first year after trauma; six of those died within 30 days after trauma. In addition, nine patients died before the second assessment 10–15 years after trauma. The remaining group of 29 patients were available for the second assessment performed with patients (*n* = 22), relatives (*n* = 2), a caregiver (*n* = 1), or a patient and caregiver (*n* = 1). Three patients were not available for the second assessment, but they were alive (GOS 2–5) according to the Swedish Tax Agency and are included in the results of GOS 1 (dead) versus GOS 2–5 (alive).

**Fig. 1:**
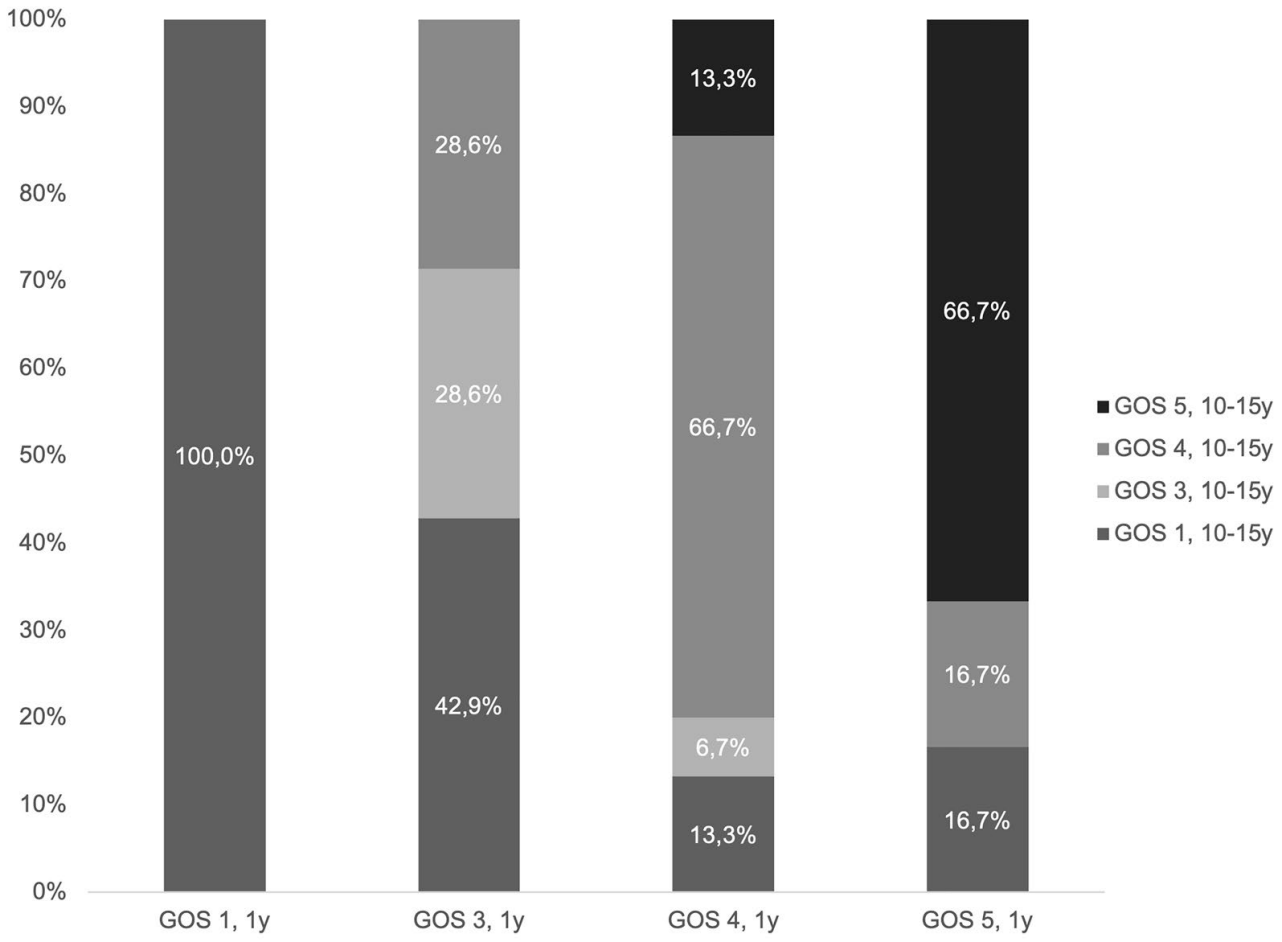
Changes in GOS to 10–15 years after trauma by GOS at 1 year. Each bar represents the GOS at 1 year. Within each bar, the distribution of GOS classifications at 10–15 years after trauma is shown (in percent). *GOS* Glasgow Outcome Scale

Most with GOS 2–5 (surviving *n* = 38) at 1 year had the same GOS 10–15 years after injury (47%, *n* = 18), whereas 29% (*n* = 11) declined, 16% (*n* = 6) improved, and 8% (*n* = 3) were missing. The GOS category at 10–15 years after trauma within each GOS category at 1 year post trauma is shown in Fig. 1.

Age correlated significantly with GFAP and GOS at 1 year and 10–15 years after trauma. Pretrauma existing neurological diseases (epilepsy, *n* = 5; transient ischemic attack, *n* = 1; previous trauma to the head, *n* = 2) correlated with GOS 10–15 years after trauma (Table 2). All analyses of 1-year outcome are adjusted for age, whereas those relating to the 10–15-year outcome are adjusted for both age and pretrauma existing neurological diseases. However, the number of patients (*n* = 9) who had GOS 1 (dead) 1 year after trauma did not allow for any adjustment of the regression analysis. No other confounder was found or needed to be adjusted for. Patient rehabilitation correlated significantly with NfL, GFAP, and GOS at 1 year and GOS at 10–15 years. Rehabilitation per se will not affect the acute-phase biomarkers and is thus considered an effect modifier and not a confounder, and as a result, it has not been adjusted for.

**Table 2:** Correlations between potential confounders, NFL and GFAP and outcome one year and 10–15 years after trauma

	GOS 1 year after trauma			GOS 10–15 years after trauma			NfL (μg/L)			GFAP (μg/L)		
	Spearman’s correlation coefficient	*p*-value	*n*	Spearman’s correlation coefficient	*p*-value	*n*	Spearman’s correlation coefficient	*p*-value	*n*	Spearman’s correlation coefficient	*p*-value	*n*
Age at time of trauma	− 0.30	0.039	47	− 0.46	0.0019	44	0.18	0.22	47	0.34	0.019	47
Sex	− 0.10	0.50	47	− 0.027	0.86	44	0.36	0.012	47	0.32	0.028	47
Neurological diseases	− 0.082	0.59	45	− 0.33	0.033	42	0.16	0.30	45	0.26	0.085	45
Diabetes mellitus	− 0.31	0.03	47	− 0.24	0.12	44	0.26	0.07	47	0.16	0.27	47
Heart diseases	− 0.21	0.16	47	− 0.34	0.022	44	0.034	0.82	47	0.011	0.94	47
High blood pressure	− 0.12	0.43	47	− 0.053	0.73	44	0.12	0.41	47	0.12	0.44	47
Marshall CT classification	− 0.095	0.53	46	− 0.16	0.32	43	0.25	0.092	46	0.19	0.20	46
Rehabilitation	− 0.66	< .0001	45	− 0.50	0.0006	43	0.43	0.0036	45	0.40	0.0061	45
Type of injury	0.070	0.64	47	0.23	0.14	44	− 0.16	0.28	47	− 0.0064	0.97	47

Spearman’s correlation coefficient was calculated on the logarithmic value of NfL and GFAP, using the highest measured value of NfL and GFAP. To be classified as a confounder, a variable need to relate to NfL and/or GFAP and GOS one year and /or GOS 10–15 years after trauma. Rehabilitation is not a confounder but an effect modifier. Age, NfL and GFAP are continuous variables, Marshall CT classification (6-grade) and GOS (5-grade) are ordinal, all other variables are dichotomous (Yes/No) and type of injury (isolated head injury or multi trauma). Neurological diseases, diabetes mellitus, high blood pressure and heart diseases are pre-trauma. CSF: Cerebral Spinal Fluid, GFAP: Glial Fibrillary Acidic Protein, GOS: Glasgow Outcome Scale, *n*: number of observations, NfL: Neurofilament Light

### Concentration Dynamics of NfL and GFAP in CSF After an sTBI

**Fig. 2:**
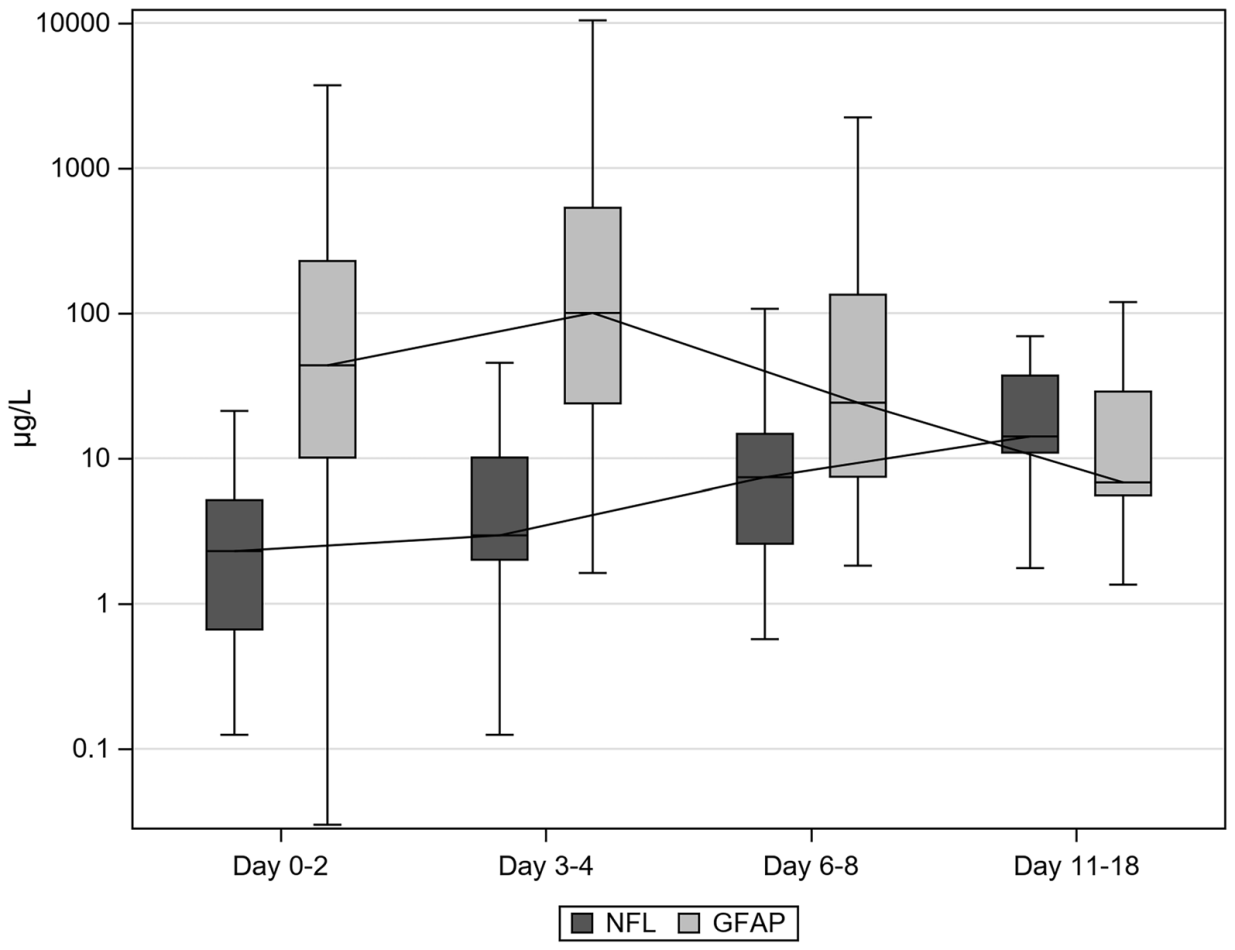
Trajectory profile of NfL and GFAP (μg/L) in CSF. Concentrations of NfL and GFAP in the CSF are shown per sample period in boxplots. The median concentrations per sample period are connected to illustrate the trajectory profile of each biomarker. Concentrations of NfL and GFAP are presented in μg/L and on a log10 scale. The sample period is expressed in days after trauma. The numbers of samples were as follows: days 0–2 (*n* = 20), days 3–4 (*n* = 32), days 6–8 (*n* = 32), and days 11–18 (*n* = 13). CSF cerebral spinal fluid, GFAP glial fibrillary acidic protein, NfL neurofilament light

CSF was analyzed on days 0–2 (*n* = 20), days 3–4 (*n* = 32), days 6–8 (*n* = 32), and days 11–18 (*n* = 13). NfL concentrations gradually increased from days 0–2 to 11–18 post injury, whereas GFAP concentrations peaked on days 3–4, after which they declined (Fig. 2).

### Initial Maximum CSF Concentrations of NfL and GFAP and Outcome 1 Year After an sTBI: GOS 1–3 (Poor) Versus GOS 4–5 (Good) Outcome

**Fig. 3:**
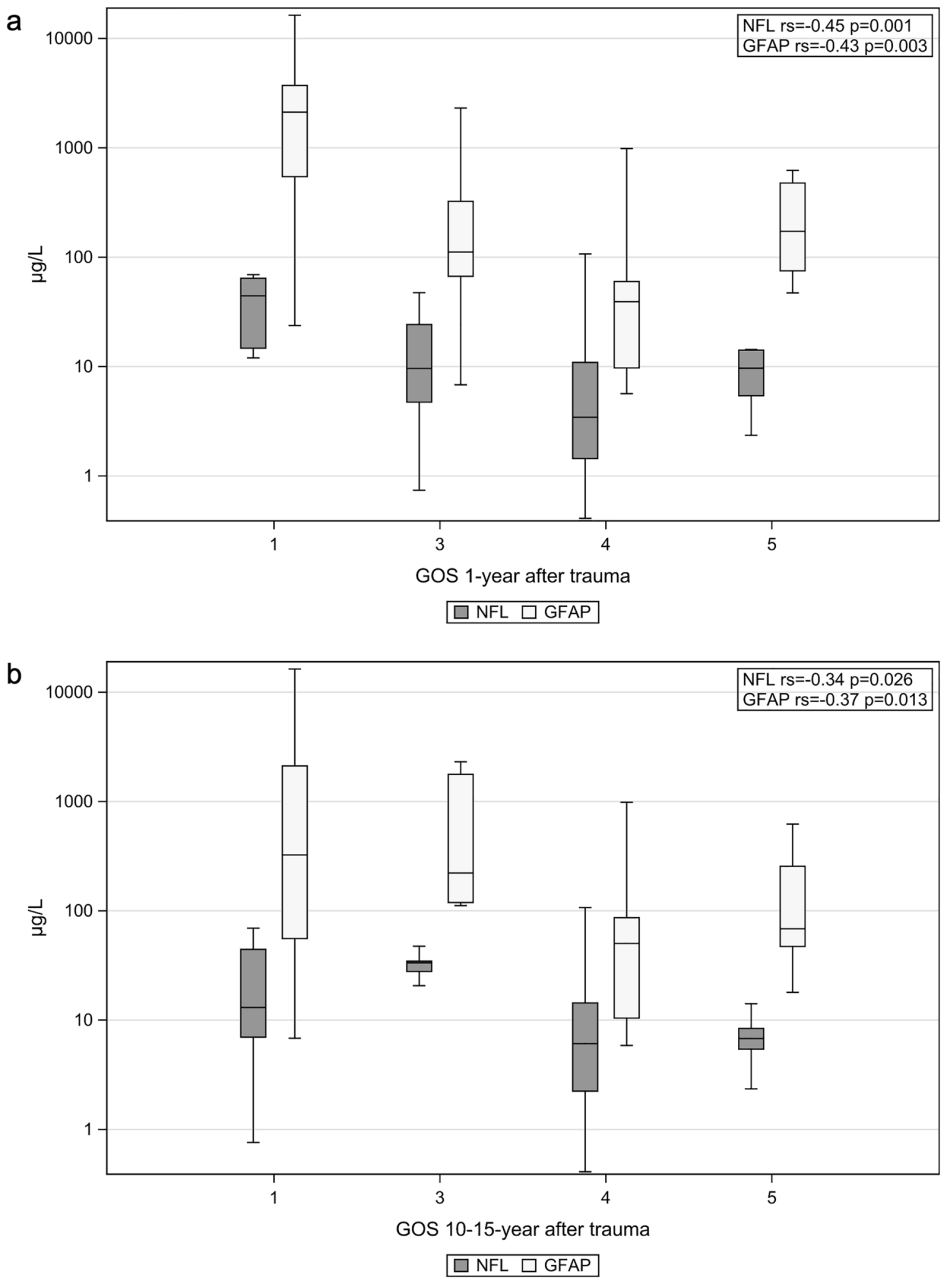
Concentrations of NfL and GFAP in each GOS category. Boxplot presenting initial CSF concentrations of NfL and GFAP in each GOS category 1–5. **a** GOS 1 year after trauma. **b** GOS 10–15 years after trauma. Each patients maximum CSF concentration of NfL and GFAP was used in the calculation. *CSF* cerebral spinal fluid, *GFAP* glial fibrillary acidic protein, *GOS* Glasgow Outcome Scale, *NfL* neurofilament light

**Fig. 4:**
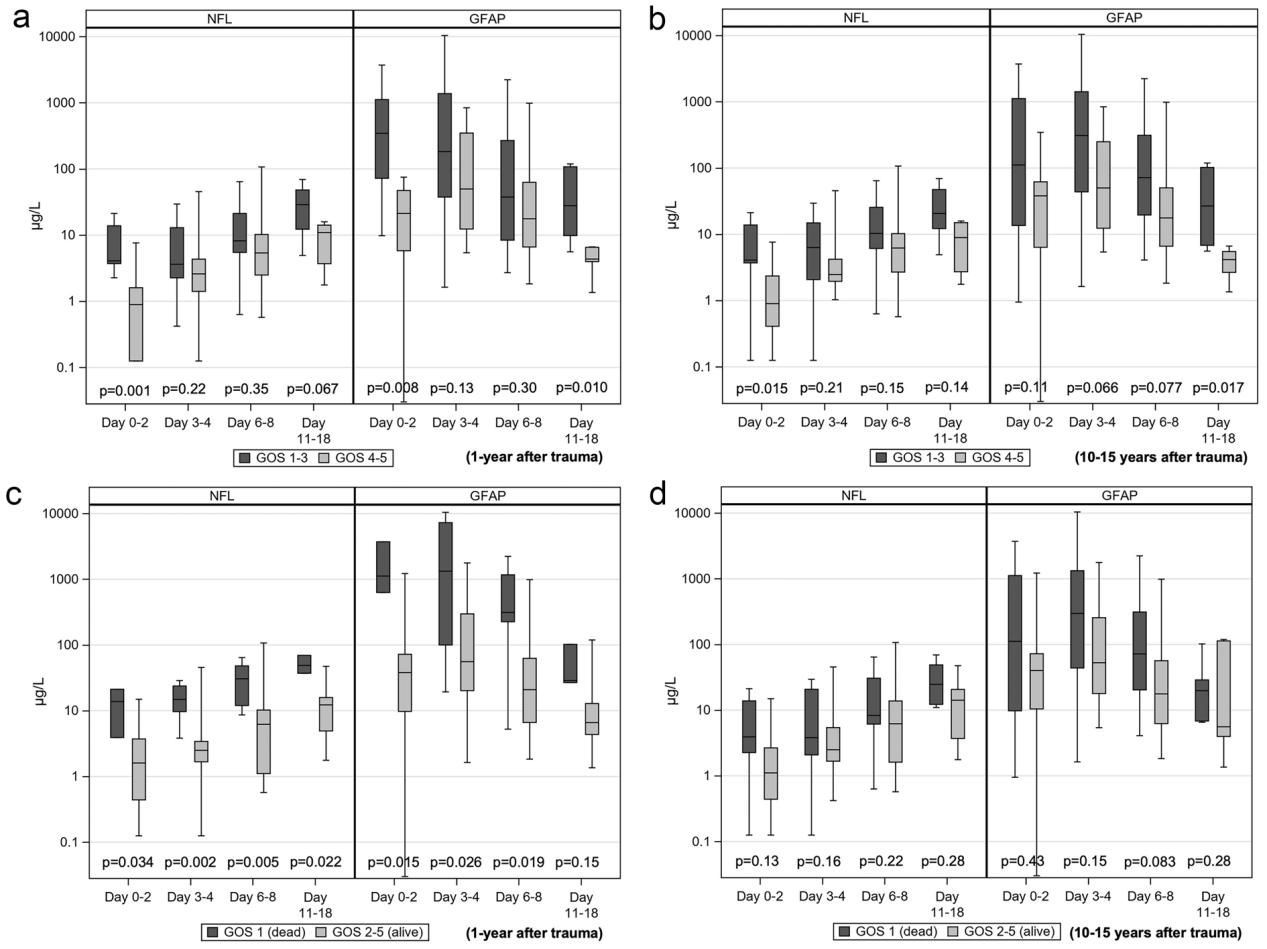
Concentrations of NfL and GFAP in those with GOS 1–3 (poor) versus those with GOS 4–5 (good outcome) and those with GOS 1 (dead) versus those with GOS 4–5 (alive) 1 year and 10–15 years after trauma. Boxplot presenting initial CSF concentrations of NfL and GFAP (μg/L on a log10 scale), separately, per sample period. Concentrations of NfL and GFAP in CSF, in stratified groups: GOS 1–3 versus GOS 4–5 outcome 1 year after trauma (**a**) and 10–15 years after trauma (**b**) and GOS 1 versus GOS 2–5 1 year after trauma (**c**) and 10–15 years after trauma (**d**). Each patients maximum CSF concentration of NfL and GFAP was used in the calculation. *CSF* cerebral spinal fluid, *GFAP* glial fibrillary acidic protein, *GOS* Glasgow Outcome Scale, *NfL* neurofilament light

Both NfL and GFAP correlated with 1-year GOS 1–5 (*r*_s_ =  − 0.45, *p* = 0.001, and *r*_s_ =  − 0.43, *p* = 0.003; adjusted for age: *r*_s_ =  − 0.42, *p* = 0.003, and *r*_s_ =  − 0.36, *p* = 0.013). Concentrations of NfL and GFAP per GOS category 1 year after trauma are shown in Fig. 3a. Levels of NfL and GFAP were significantly higher in patients with GOS 1–3 than in those with GOS 4–5 before and after adjustment for age (Table 3). Patients with GOS 4–5 had (adjusted fold change) 62% lower NfL concentrations and 76% lower GFAP concentrations than patients with GOS 1–3 outcome 1 year after trauma (Table 3). We found significantly higher levels of NfL on days 0–2 and of GFAP on days 0–2 and 11–18 in patients with GOS 1–3 than in patients with GOS 4–5 (Fig. 4a).

**Table 3:** Comparison of initial NfL and GFAP concentrations between poor versus good outcome and dead versus alive one year and 10–15 years after trauma

Variable	GOS 1–3 (poor outcome)	GOS 4–5 (good outcome)	Unadjusted		Adjusted		GOS 1 (Dead)	GOS 2–5 (Alive)	Unadjusted		Adjusted	
			*p*-value*	Fold change (95% CI)	*p*-value	Fold change (95% CI)			*p*-value*	Fold change (95% CI)	*p*-value	Fold change (95% CI)
1 year after trauma												
NFL (μg/L)	14.73 (6.3–34.6)	5.69 (2.2–14.1)	0.011	0.35 (0.16–0.76)	0.019	0.38 (0.17–0.85)	44.37 (14.7–64.2)	6.65 (2.9–14.3)	< .001	0.19 (0.07–0.46)	0.002	0.19 (0.07–0.54)
	*n* = 25	*n* = 22					*n* = 9	*n* = 38				
GFAP (μg/L)	172.50 (86.5–1771.0)	51.45 (18.0–89.5)	0.003	0.19 (0.06–0.53)	0.010	0.24 (0.08–0.70)	2116.00 (544.5–3717.0)	68.90 (38.1–221.7)	< .001	0.06 (0.02–0.22)	< .001	0.09 (0.02–0.34)
	*n* = 25	*n* = 22					*n* = 9	*n* = 38				
10–15 years after trauma												
NFL (μg/L)	20.64 (10.9–44.4)	6.09 (3.2–14.1)	0.005	0.34 (0.16–0.72)	0.031	0.36 (0.14–0.91)	13.05 (7.0–44.4)	6.32 (3.2–15.9)	0.074	0.47 (0.20–1.07)	0.38	0.62 (0.21–1.81)
	*n* = 23	*n* = 21					*n* = 18	*n* = 29				
GFAP (μg/L)	306.90 (100.0–2116.0)	57.82 (38.1–86.5)	0.003	0.17 (0.06–0.50)	0.023	0.24 (0.07–0.82)	324.25 (55.7–2116.0)	62.70 (38.1–119.2)	0.011	0.23 (0.07–0.71)	0.19	0.42 (0.11–1.59)
	*n* = 23	*n* = 21					*n* = 18	*n* = 29				

Data are presented as the median (IQR) for numeric variables. *Fisher’s Non-Parametric Permutation test was used to compare concentrations of NfL and GFAP between poor vs good outcome and dead vs alive; otherwise ANCOVA was used. Adjusted for age one year after trauma and age and neurological diseases 10–15 years after trauma. Each patients maximum CSF concentration of NfL and GFAP was used in the calculation. *CI* confidence interval, *CSF* Cerebral Spinal Fluid, *GFAP* Glial Fibrillary Acidic Protein, *GOS* Glasgow Outcome Scale, *IQR* interquartile range *n* number of observations, *NfL* Neurofilament Light

**Fig. 5:**
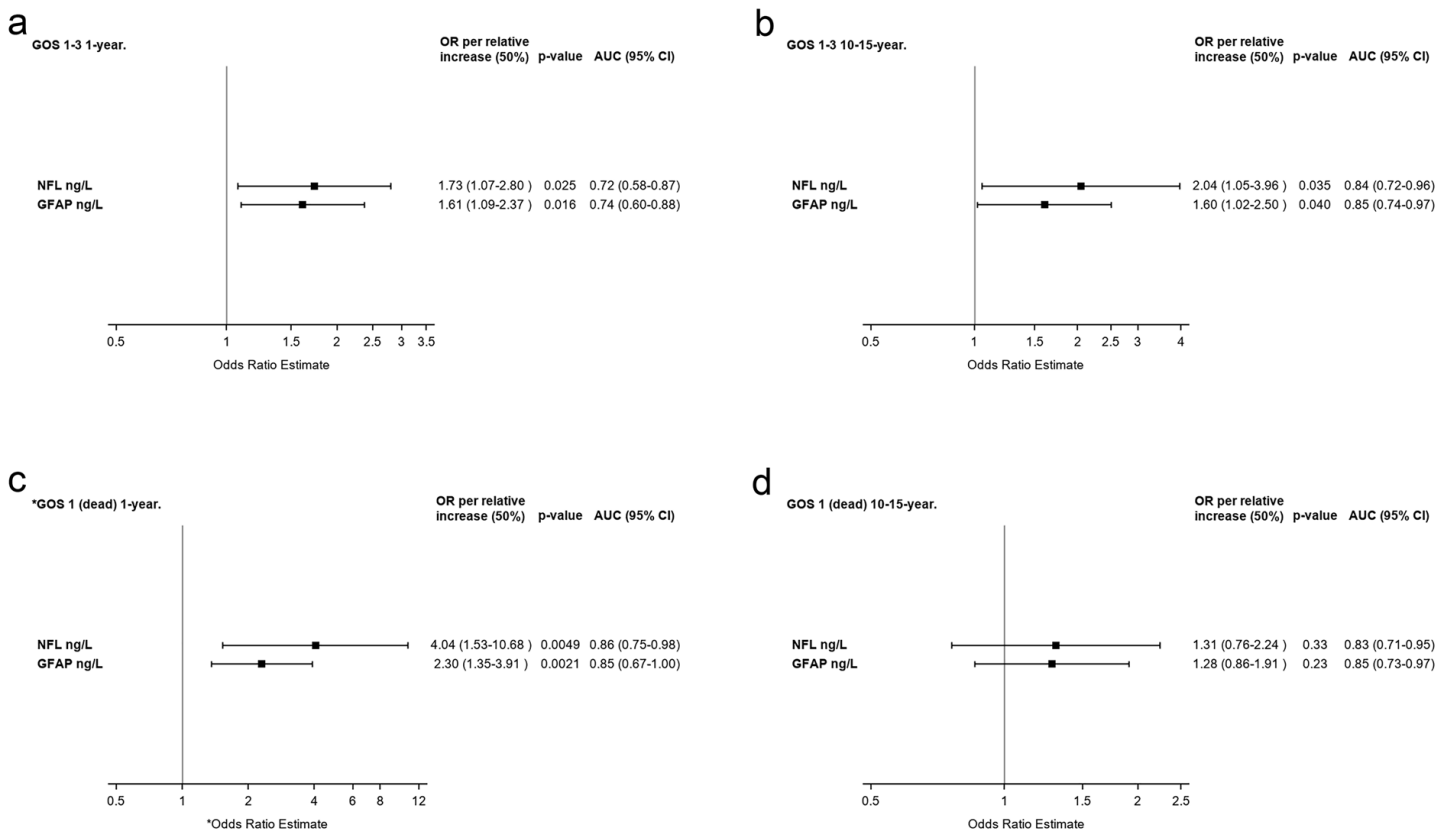
The OR for GOS 1–3 (poor) outcome and GOS 1 (dead) 1 year and 10–15 years after trauma by NfL and GFAP. The OR is the ratio of the odds for the dependent variable of GOS 1–3 outcome or GOS 1 with a relative increase in initial NfL or GFAP in the CSF of 50%. **a** GOS 1–3 outcome 1 year after trauma. **b** GOS 1–3 outcome 10–15 years after trauma. **c** GOS 1 outcome 1 year after trauma. **d** GOS 1 10–15 years after trauma. *The analysis was unadjusted due to few events. GOS 1–3 outcome at 1 year is adjusted for age. GOS 1–3 outcome and GOS 1 10–15 years after trauma are adjusted for age and neurological diseases. The results for OR, *p*-value and area under the ROC curve (AUC) are based on original values and not stratified groups. Each patients maximum CSF concentration of NfL and GFAP was used in the calculation. *CSF* cerebral spinal fluid, *GFAP* glial fibrillary acidic protein, *GOS* Glasgow Outcome Scale, *NfL* neurofilament light

**Fig. 6:**
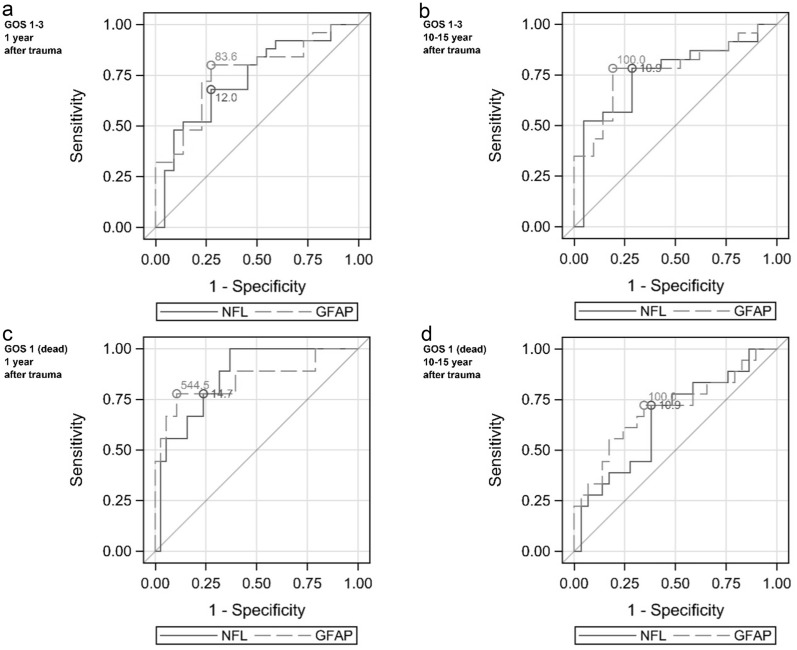
Receiver operating characteristic curve (ROC) for NfL and GFAP in the CSF for GOS 1–3 outcome and GOS 1 outcome. ROC illustrating the quality at which specific levels of NfL and GFAP in the CSF can separate GOS 1–3 from GOS 4–5 outcome and GOS 1 outcome from GOS 2–5 outcome. **a** GOS 1 outcome 1 year after trauma. **b** GOS 1–3 outcome 10–15 years after trauma. **c** GOS 1 outcome 1 year after trauma. **d** GOS 1 10–15 years after trauma. The marked cut-off value is calculated using the sum of best sensitivity and specificity for each biomarker. Each patients maximum CSF concentration of NfL and GFAP was used in the calculation. *CSF* cerebral spinal fluid, *GFAP* glial fibrillary acidic protein, *GOS* Glasgow Outcome Scale, *NfL* neurofilament light

After age adjustment, a relative increase in NfL and GFAP of 50% significantly increased the odds of GOS 1–3 outcome 1 year after trauma (OR 1.73, *p* = 0.025, and OR 1.61, *p* = 0.016, respectively; Fig. 5a). To find cutoff values for the 1-year outcome, we used the Youden index and noted that an NfL concentration of ≥ 12.0 μg/L (Youden index, *J* = 0.41) separated patients with GOS 1–3 outcome from those with GOS 4–5 outcome with a sensitivity of 0.68 and a specificity of 0.73. Similarly, a GFAP concentration of ≥ 83.6 μg/L (*J* = 0.53) separated patients with GOS 1–3 outcome from those with GOS 4–5 outcome with a sensitivity of 0.80 and a specificity of 0.73 (Fig. 6a).

### Initial Maximum CSF Concentrations of NfL and GFAP and Outcome 10–15 Years After an sTBI: GOS 1–3 (Poor) Versus GOS 4–5 (Good) Outcome

Both NfL and GFAP correlated with 10–15-year GOS 1–5 (*r*_s_ =  − 0.34, *p* = 0.026, and *r*_s_ =  − 0.37, *p* = 0.013), but after adjustment for age and neurological diseases, the correlation was lost (*r*_s_ =  − 0.27, *p* = 0.088, and *r*_s_ =  − 0.24, *p* = 0.13). Concentrations of NfL and GFAP per GOS category at 10–15 years after trauma are shown in Fig. 3b. Patients with GOS 1–3 outcome 10–15 years after trauma had significantly higher concentrations of both NfL and GFAP than those with GOS 4–5 outcome. After adjustment for age and neurological diseases, patients with GOS 1–3 outcome had significantly higher concentrations of NfL and GFAP (Table 3). Patients with GOS 4–5 outcome had (adjusted fold change) 64% lower NfL and 76% lower GFAP concentrations than patients with GOS 1–3 outcome 10–15 years after trauma (Table 3). We found significantly higher levels of NfL on days 0–2 and of GFAP on days 11–18 in patients with GOS 1–3 outcome than in those with GOS 4–5 outcome (Fig. 4b). After adjustments for age and neurological diseases, a relative increase in NfL and GFAP of 50% significantly increased the odds of a GOS 1–3 outcome 10–15 years after trauma (OR 2.04, *p* = 0.035, and OR 1.60, *p* = 0.040, respectively; Fig. 5b). At levels ≥ 10.9 μg/L (*J* = 0.49), NfL separated patients with a GOS 1–3 outcome from those with a GOS 4–5 outcome with a sensitivity of 0.78 and a specificity 0.71, whereas at levels ≥ 100 μg/L (*J* = 0.59), GFAP separated patients with a GOS 1–3 outcome with a sensitivity of 0.78 and a specificity of 0.81 (Fig. 6b).

### Initial Maximum CSF Concentrations of NfL and GFAP and Mortality at 1 Year and 10–15 Years After an sTBI: GOS 1 (Dead) Versus GOS 2–5 (Alive)

Patients who died (GOS 1) within 1 year after trauma had significantly higher levels of NfL and GFAP than survivors (GOS 2–5) before and after adjustments for age (Table 3). Patients with GOS 2–5 outcome had (adjusted fold change) 81% lower NfL concentrations and 91% lower GFAP concentrations than patients with GOS 1 outcome 1 year after trauma (Table 3). We found significantly higher levels of NfL on days 0–2, 3–4, 6–8, and 11–18 and of GFAP on days 0–2, 3–4, and 6–8 in patients with GOS 1 compared with those with GOS 2–5 outcome (Fig. 4c). The number of patients with GOS 1 within 1 year was too low to allow adjustment analysis. Unadjusted, a relative increase in NfL and GFAP concentrations of 50% significantly increased the OR for dying (GOS 1) (OR 4.04, *p* = 0.0049, and OR 2.30, *p* = 0.0021, respectively; Fig. 5c). An NfL concentration of ≥ 14.7μg/L (*J* = 0.54) was able to separate patients with GOS 1 within 1 year after trauma with a sensitivity of 0.78 and a specificity of 0.76. At a concentration of 545 μg/L (*J* = 0.67), GFAP separated patients with GOS 1 within 1 year with a sensitivity of 0.78 and a specificity of 0.89 (Fig. 6c). Further, patients with GOS 1 within 10–15 years after trauma had significantly higher concentrations of NfL and GFAP than those with GOS 2–5. However, after adjustment for age and neurological diseases, this difference was lost (Table 3). There were no significant concentration differences in NfL or GFAP between patients with GOS 1 or GOS 2–5 outcome 10–15 years after trauma per sample period (Fig. 4d). Finally, a relative increase in NfL or GFAP concentrations was not significantly associated with mortality within 10–15 years after trauma, neither before nor after adjustments for age and neurological diseases (Fig. 5d).

## Discussion

This study demonstrated that concentrations of CSF NfL gradually increased during the first 2 weeks after an sTBI, whereas CSF GFAP peaked during the initial days. High CSF concentrations of both biomarkers were associated with a GOS 1–3 (poor) outcome at 1 year and 10–15 years after trauma and only GOS 1 (dead) at 1 year but not 10–15 years after trauma.

The gold standard for evaluating brain injury severity is the GCS and CT images. These clinical variables, combined with age and pupil reactivity, among others, are used in a prognostic model developed by the IMPACT investigators to estimate 6-month outcome after a moderate to severe TBI [22]. Adding biomarkers, such as NfL and GFAP in serum, in models similar to International Mission for Prognosis and Analysis of Clinical Trails in TBI (IMPACT) has revealed improved predictive properties [6, 23, 24]. It is clinically important to identify and study objective factors, such as biomarkers, as these may help us to better evaluate the initial brain damage and further provide an early indication of the prognosis after an sTBI. This may lead to better individualized treatment and help direct hospital resources.

The presence and concentration of blood-based biomarkers in patients with TBI is being increasingly investigated but less so in the CSF. Although CSF and serum concentrations of NfL and GFAP cannot be compared completely because of passage over an injured BBB, the influence of the glymphatic system, and blood clearance, among others, our results appear to agree with studies of NfL and GFAP in serum. Similar to the NfL concentration noted over time in our population, the trajectory profile of NfL after TBI has previously been shown to increase gradually over time in both serum and the CSF [23, 24]. One study included part of our cohort [6]. GFAP concentrations in serum have previously been described to peak within 16–48 h after a TBI [9, 23, 25]. Similarly, we found that GFAP in the CSF peaked during the initial days but later than 48 h after trauma. It is possible to speculate that the later peak in the CSF, as noted in the present study, may be explained by the restoration of the disrupted BBB within 24 h post injury, as shown by others [26, 27], and the continuous accumulation in the CSF due to injured and reactive astrocytes [2]. Further, other studies suggest that a TBI disrupts the brain clearance by a reduced and impaired glymphatic function [28, 29].This may also lead to an earlier decrease of concentrations in the blood.

To be able to investigate whether concentrations of NfL and GFAP in the CSF relate to outcome 1 year and 10–15 years after trauma, we considered different confounders. We considered age and pretrauma existing neurological diseases to be confounding factors. After adjustment for age, we found that both NfL and GFAP in the CSF relate to 1-year outcome. Al Nimer et al. [24] found that unadjusted CSF NfL related to 6–12-month outcome after trauma; however, after adjustments for clinical variables, such as age, GCS, pupil response, and CT, the relationship was lost. Several studies have previously found that NfL and GFAP in serum relate to 3-month, 6-month, and 1-year outcome [6, 9, 23, 24, 30, 31]; two of them included patients from the present cohort [6, 9]. In agreement with our results in the CSF, NfL and GFAP in serum have been shown by others to be able to discriminate between a poor and a good outcome [30, 31]. One study included patients from our cohort [6]. Further, we found NfL and GFAP to relate to outcome 10–15 years trauma, adjusted for age and neurological disease. To our knowledge, we are the first to study how initial concentrations of NfL and GFAP in the CSF relate to outcome as long as 10–15 years after trauma. Patients with TBI can have elevated concentrations of NfL and GFAP in serum for month to years compared with controls [32, 33]. Newcombe et al. [33] found that higher levels of NfL in patients with TBI was related to worsening in functional outcome (Glasgow Outcome Scale Extended) between 8 months and > 5 years after trauma. Further, NfL levels in the chronic phase after a TBI have been found to be associated with brain volume loss on magnetic resonance imaging years following TBI, whereas GFAP did not [32, 33]. In contrast to these studies, we studied the initial acute concentrations of NfL and GFAP in CSF and not serum. We have previously shown that GOS at 1 year correlates with GOS 10–15 years after trauma, although individual improvement and deteriorations were noted [15]. The continuous relation between the acute-phase concentrations of NfL and GFAP in CSF and outcome 10–15 years after trauma is possibly a reflection of the long-lasting effect the initial injury has on function. In contrast, subacute and chronic levels may indicate ongoing processes and an association with later deterioration [32, 33].

When unadjusted, our data showed that CSF concentrations of NfL and GFAP related to mortality 1 year after trauma. However, when adjusted for age and neurological disease, NfL and GFAP did not relate to mortality at 10–15 years after trauma. Previous studies, two including some patients from the present study [6, 9], showed that NfL and GFAP in serum related to mortality 1 year after trauma [6, 9, 31]. McMillan et al. [34] found that up to 13 years after trauma, patients with sTBI had higher mortality, but the cause of death itself was similar to that in control patients. This suggests that factors other than the initial brain injury may influence late deaths after a TBI and may thus explain part of our results.

Clinically, biomarkers in blood are preferable, being easier to access and less invasive. Today, intraparenchymal pressure probes are gaining in popularity in ICP monitoring, making biomarkers in blood even more important. Several investigators have previously studied the prognostic abilities of NfL and GFAP in blood, but few have done so in the CSF [35]. In this study, intraventricular catheters were used for ICP monitoring, enabling us to analyze biomarkers in the CSF and contributing to the knowledge of how these biomarkers relate to long-term outcome, with the advantage of being analyzed in close proximity to the actual pathophysiological processes (i.e., trauma) [36]. Further, in a previous study by Shahim et al. [6], we compared levels of NfL in serum and CSF in study participants who were part of this patient cohort. This investigation demonstrated a correlation between trajectory CSF and serum levels over 12 days, with CSF levels being 100-fold higher than those noted in serum [6]. Thus, because CSF catheters are more rarely used, it seems appropriate to analyze serum NfL with the novel, more precise techniques.

Advantages in proteomic by employing techniques such as mass spectrometry in which multiple proteins can be analyzed simultaneously will improve our knowledge regarding the heterogenic pathophysiological mechanism involved after a TBI. Studying these large data sets will help find more specified biomarkers for diagnosis, injury progression, prognosis, and possible therapy targets in the future [37]. Today, mass spectrometry-based proteomics studies highlight the inflammatory response and give new insight to pathophysiological pathways after a TBI [38–40]. Meanwhile, studies like ours will be part of the foundation of knowledge helping to implement current knowledge into clinical practice.

This study is limited by its single-center design and small sample set, restricting the interpretation of our results. Patients from the entire southwestern part of Sweden who have an sTBI are transferred and treated at the NICU at Sahlgrenska University Hospital. However, patients deteriorating at the primary hospital are not transferred to the NICU, producing an unavoidable selection bias. At the NICU, children were also treated and included in the study (*n* = 6). Statistical analyses were performed with and without the children. Without the pediatric population, the statistical relation between NfL and GFAP in CSF and the 1-year and 10–15-year outcome was enhanced. A possible explanation may be the almost inverted baseline temporal profiles of these biomarkers in healthy children [41, 42]. Further, the recovery process and the pathophysiological mechanisms after a TBI may also differ from that of an adult [43]. Studies of pediatric traumatic brain injury and the prediction of outcome with biomarkers are scares. Therefore, the results including the children are presented in the Supplementary Material together with descriptive data of the pediatric population.

CSF samples were only collected when the attending neurosurgeon regarded the ICP as reasonably stable. As a result, some samples could not be obtained, which is an eventual selection bias. Because the CSF was collected intermittently, with some missing samples, we chose to use the maximum concentrations of NfL and GFAP instead of a specific time to calculate the receiver operating characteristic curve. We chose not to exclude outliers from our data. However, we used log transformation and nonparametric statistics. Because of the lack of CSF, 46 patients were excluded from this study. These missing data may affect our generalizability. However, the population included in this study is similar to the original population with regard to age, sex, and 1-year outcome [15]. Three patients (6%) did not participate in the second assessment. However, they were still alive at the second follow-up and are thus included in the analysis of being dead or alive. We did not use imputation and, if it is associated with exposure or outcome, it may have affected our results and the generalizability of the study. As previously discussed, we adjusted for age as a confounding factor. We also adjusted for pretrauma neurological diseases as a confounding factor, although the statistical significance for this factor disappeared when we omitted the pediatric population. We did not adjust for specific lesion types, locations, and severities. The Marshall score is an approximate and does not replace individual and aggregated information on specific intracranial lesion types.

Assuming that the brain damage and the recovery process would be stable 10–15 years after trauma, the second assessment was performed. To minimize loss to the second follow-up, the GOS interviews were held by phone. Finally, both interviewers assessing GOS were blinded to NfL and GFAP concentrations, but at the second assessment, the interviewers were not blinded to the 1-year neurological evaluation. We used the five-graded version of the GOS because it has higher agreement between investigators than the eight-graded Glasgow Outcome Scale Extended [20].

## Conclusions

This study demonstrates that high concentrations of NfL and GFAP in CSF relate to a poorer outcome 1 year and 10–15 years after an sTBI. These results are in agreement with studies performed in serum at 1 year. Further, we found trajectory CSF profiles of both biomarkers. Our findings further contribute to our understanding of how biomarkers relate to both outcome and the initial injury. To implement prognostic markers in clinical practice as biomarkers of both prognosis and injury severity, we need larger studies to test and validate these biomarkers in models and studies designed to better define injury severity.

## Supplementary Information

Below is the link to the electronic supplementary material.

Supplementary file1 (DOCX 786 KB)

Supplementary file2 (DOCX 3796 KB)

## Clinical Trial Registration

ClinicalTrials.gov identifier NCT05138692.
