# Supplementary material for: Acute-Phase Neurofilament Light and Glial Fibrillary Acidic Proteins in Cerebrospinal Fluid Predict Long-Term Outcome After Severe Traumatic Brain Injury
Source: Neurocrit Care. 2024 May 20;41(3):813–27. doi: 10.1007/s12028-024-01998-0 (PMC11599393; doi:10.1007/s12028-024-01998-0)
Supplement: Supplementary file 1 — Supplementary file1 (DOCX 786 KB) [file 12028_2024_1998_MOESM1_ESM.docx]

Supplement 1.

Pediatric population

| Supplement 1: Table 1. Patient Characteristics – Children | | |
| --- | --- | --- |
| Variable | | Total |
| Included patients, n (%) | | 6 (100.0%) |
| Age at time of trauma, mean (SD), median (min-max) | | 13.5 (3.25) |
|  |  | 15.5.1 (8; 16) |
| Sex, n (%) | |  |
| Male | | 4 (66.7%) |
| Female | | 2 (33.3%) |
| Pre-trauma diseases, n (%) | |  |
| Neurological disease | | 0 (0.0%) |
| Diabetes mellitus | | 2 (33.3%) |
| Neurosurgery, n (%) | |  |
| Evacuation of hematomas | | 2 (33.3%) |
| Evacuation of hematomas and decompressive craniectomy | | 1 (16.7%) |
| Decompressive craniectomy | | 2 (33.3%) |
| Revision of skull fracture | | 1 (16.7%) |
| Dura reconstruction | | 1 (16.7%) |
| Type of trauma, n (%) | |  |
| Isolated head trauma | | 2 (33.3%) |
| Multi trauma | | 4 (66.7%) |
| Cause of trauma, n (%) | |  |
| Road traffic accident | | 5 (83.3%) |
| Fall | | 1 (16.7%) |
| Marshall CT-Classification, n (%) | |  |
| Diffuse injury I (Normal) | | 0 (0.0%) |
| Diffuse injury II (Lesions, present cisterns, midline shift 0-5mm) | | 1 (16.7%) |
| Diffuse injury III (Lesions, cisterns compressed, midline shift 0-5mm) | | 2 (33.3%) |
| Diffuse injury IV (Midline shift > 5 mm) | | 3 (50.0%) |
| V (Any surgically evacuated lesion) | | 0 (0.0%) |
| VI (Non evacuated mass lesion, >25cc lesion) | | 0 (0.0%) |
| Rehabilitation, n (%) | | 4 (66.7%) |
| Glasgow Outcome Scale (GOS), n (%) | |  |
| 1-yer after trauma | |  |
| GOS 1-3 (Poor outcome) | GOS 1 (Dead) | 0 (0.0%) |
|  | GOS 2 (Vegetative state) | 0 (0.0%) |
|  | GOS 3 (Severe disability) | 1 (16.7%) |
| GOS 4-5 (Good outcome) | GOS 4 (Moderate disability) | 0 (0.0%) |
|  | GOS 5 (Good recovery) | 5 (83.3%) |
| 10-15 years after trauma | |  |
| GOS 1-3 (Poor outcome) | GOS 1 (Dead) | 2 (33.3%) |
|  | GOS 2 (Vegetative state) | 0 (0.0%) |
|  | GOS 3 (Severe disability) | 0 (0.0%) |
| GOS 4-5 (Good outcome) | GOS 4 (Moderate disability) | 0 (0.0%) |
|  | GOS 5 (Good recovery) | 4 (66.7%) |
| Patient characteristics presented for the included children. For categorical variables n (%) is presented. For continuous variables Mean (SD) / Median (Min; Max) is presented. GOS: Glasgow Outcome Scale, n: number of observations. | | |

| Supplement 1: Table 2. Concentrations of NfL and GFAP in CSF by sample period in the pediatric population | | | | | | | | |
| --- | --- | --- | --- | --- | --- | --- | --- | --- |
| Sample period | Biomarker | Number of observations | Median | Lower Quartile | Upper Quartile | Minimum | Maximum | Standard Deviation |
| Day 0-2 | NFL | 4 | 0.34 | 0.15 | 0.54 | 0.13 | 0.59 | 0.23 |
|  | GFAP | 4 | 9.28 | 6.50 | 23.53 | 6.35 | 35.15 | 13.66 |
| Day 3-4 | NFL | 1 | 1.73 | 1.73 | 1.73 | 1.73 | 1.73 | . |
|  | GFAP | 1 | 11.27 | 11.27 | 11.27 | 11.27 | 11.27 | . |
| Day 6-8 | NFL | 3 | 3.05 | 1.48 | 8.61 | 1.48 | 8.61 | 3.74 |
|  | GFAP | 3 | 4.30 | 3.18 | 145.95 | 3.18 | 145.95 | 82.10 |
| Day 11-18 | NFL | 2 | 34.50 | 34.00 | 35.00 | 34.00 | 35.00 | 0.71 |
|  | GFAP | 2 | 10.63 | 8.56 | 12.70 | 8.56 | 12.70 | 2.93 |
| Concentrations of NfL and GFAP by sample day in the pediatric population, also displayed in supplement figure 2. CSF: cerebrospinal fluid, GFAP: Glial fibrillary acidic protein, NfL: Neurofilament light. | | | | | | | | |

| Supplement 1: table 3. Concentrations of NfL and GFAP by GOS 1-3 or GOS 4-5 one year and 10-15 years after trauma in the pediatric population | | | | | | | | |
| --- | --- | --- | --- | --- | --- | --- | --- | --- |
| GOS | Biomarker | Number of Observation | Median | Lower Quartile | Upper Quartile | Minimum | Maximum | Standard Deviation |
| 1 year after trauma | | | | | | | | |
| 3 | NFL | 1 | 34.00 | 34.00 | 34.00 | 34.00 | 34.00 | . |
|  | GFAP | 1 | 8.56 | 8.56 | 8.56 | 8.56 | 8.56 | . |
| 5 | NFL | 5 | 1.48 | 0.49 | 3.05 | 0.24 | 35.00 | 15.10 |
|  | GFAP | 5 | 15.96 | 6.65 | 43.66 | 6.35 | 283.30 | 119.55 |
| 10-15 years after trauma | | | | | | | | |
| 1 | NFL | 2 | 17.74 | 1.48 | 34.00 | 1.48 | 34.00 | 23.00 |
|  | GFAP | 2 | 7.46 | 6.35 | 8.56 | 6.35 | 8.56 | 1.56 |
| 5 | NFL | 4 | 1.77 | 0.37 | 19.03 | 0.24 | 35.00 | 16.92 |
|  | GFAP | 4 | 29.81 | 11.31 | 163.48 | 6.65 | 283.30 | 131.55 |
| Concentrations of NfL and GFAP in the pediatric population with GOS 1-3 or GOS 4-5 one year and 10-15 years after trauma. Each patients maximum CSF concentration of NfL and GFAP was used. Also displayed in supplement figure 3. CSF: cerebrospinal fluid, GFAP: Glial fibrillary acidic protein, GOS: Glasgow outcome scale, NfL: Neurofilament light. | | | | | | | | |

**Supplement 1: Fig 1. Changes in GOS to 10-15 years after trauma by GOS at one year in the pediatric population.**


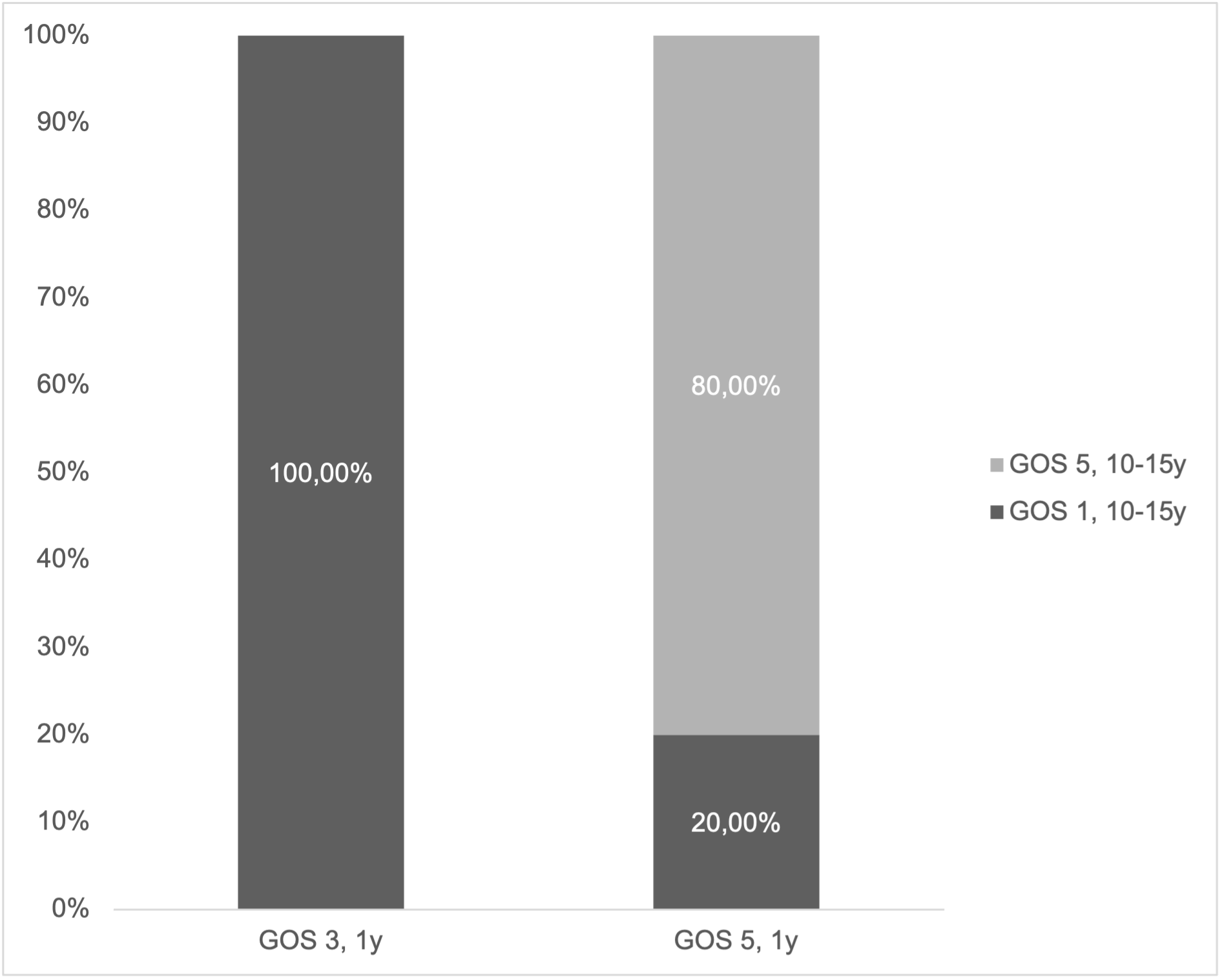


Each bar represents the GOS at one year. Within each bar, the distribution of GOS classifications at 10-15 years after trauma is shown (in percent). GOS: Glasgow Outcome Scale.

**Supplement 1: Fig 2. Trajectory profile of NfL and GFAP (μg/L) in CSF in the pediatric population.**


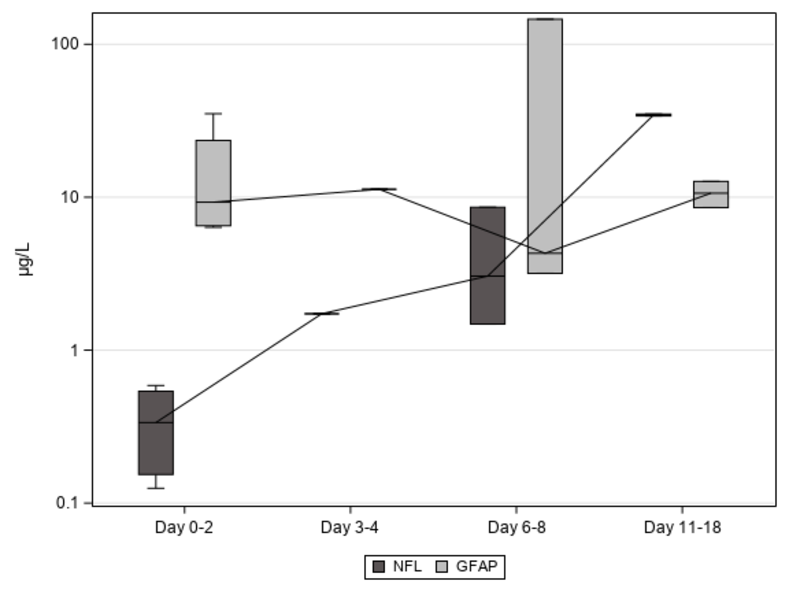


Concentrations of NfL and GFAP in the CSF are shown per sample period in boxplots. The median concentrations per sample period are connected to illustrate the trajectory profile of each biomarker. Concentrations of NfL and GFAP are presented in μg/L and on a log10 scale. The sample period is expressed in days after trauma. The number of samples were day 0-2 (n=4), day 3-4 (n=1), day 6-8 (n=3) and day 11-18 (n=2). CSF: Cerebral Spinal Fluid, GFAP: Glial Fibrillary Acidic Protein, NfL: Neurofilament Light.

**Supplement 1: Fig 3a-b. Concentrations of NfL and GFAP in those with GOS 1-3 (poor) vs. those with GOS 4-5 (good outcome) one year and 10-15 years after trauma in the pediatric population.**

**
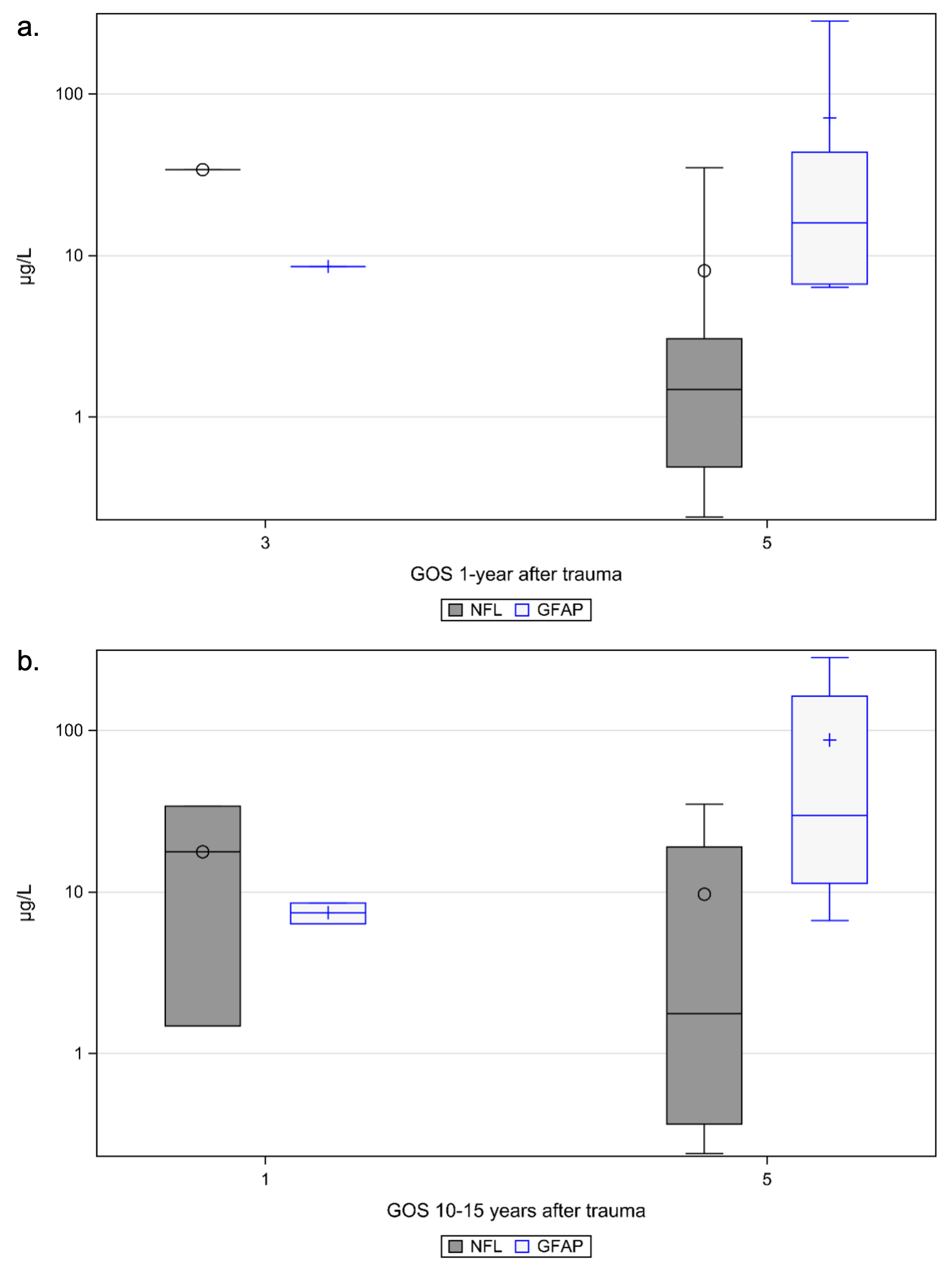
**

Boxplot presenting initial CSF concentrations of NfL and GFAP (μg/L on a log10 scale), over the whole sample period, stratified in groups: GOS 1-3 vs. GOS 4-5 outcome at a. One year after trauma b. 10-15 years after trauma. One year after trauma 1 patient where a GOS 3, 5 patients GOS 5, 10-15 years after trauma 2 patients where GOS 1 and 4 patients GOS 5. Each patients maximum CSF concentration of NfL and GFAP was used in the calculation. CSF: Cerebral Spinal Fluid, GFAP: Glial Fibrillary Acidic Protein, GOS: Glasgow Outcome Scale, NfL: Neurofilament Light.
