# Supplementary material for: Acute-Phase Neurofilament Light and Glial Fibrillary Acidic Proteins in Cerebrospinal Fluid Predict Long-Term Outcome After Severe Traumatic Brain Injury
Source: Neurocrit Care. 2024 May 20;41(3):813–27. doi: 10.1007/s12028-024-01998-0 (PMC11599393; doi:10.1007/s12028-024-01998-0)
Supplement: Supplementary file 2 — Supplementary file2 (DOCX 3796 KB) [file 12028_2024_1998_MOESM2_ESM.docx]

Supplement 2.

The numeric of the tables and figures correspond to the referred tables and figures in the text, these results include the pediatric and adult population.

| Supplement 2: Table 1. Patient characteristics |  |  |  |  |  |
| --- | --- | --- | --- | --- | --- |
| Variable |  | 1-year after trauma | | 10-15 years after trauma | |
| Included patients, n (%) | Total | Poor Outcome | Good outcome | Poor Outcome | Good outcome |
| Total | 53 (100) | 26 (100) | 27 (100) | 25 (100) | 25 (100) |
| Glasgow outcome scale (GOS), n (%) |  |  |  |  |  |
| GOS 1 (Dead) |  | 9 (35) |  | 20 (80) |  |
| GOS 2 (Vegetative) |  | 0 (0) |  | 0 (0.0) |  |
| GOS 3 (Severe disability) |  | 17 (65) |  | 5 (20) |  |
| GOS 4 (Moderate disability) |  |  | 16 (59) |  | 15 (60) |
| GOS 5 (Good recovery) |  |  | 11 (41) |  | 10 (40) |
| Sex, n (%) |  |  |  |  |  |
| Men | 43 (81) | 20 (77) | 23 (85) | 19 (76) | 21 (84) |
| Women | 10 (19) | 6 (23) | 4 (15) | 6 (24) | 4 (16) |
| Age at time of trauma, Mean (SD), median (min-max) |  | 48 (17.0) | 36 (17.9) | 50 (17.9) | 34 (16.1) |
|  | 41 (8-76) | 48 (10; 76) | 37 (8; 71) | 56 (10; 76) | 32 (8; 71) |
| Pre-trauma diseases, n (%) |  |  |  |  |  |
|  |  |  |  |  |  |
| Neurological disease, missing=2 | 8 (15) | 6 (23) | 2 (7) | 7 (28) | 1 (4) |
| Heart disease | 4 (8) | 3 (12) | 1 (4) | 4 (16) | 0 (0) |
| High blood pressure | 3 (6) | 2 (8) | 1 (4) | 2 (8) | 1 (4) |
| Diabetes mellitus | 4 (8) | 2 (8) | 2 (7) | 3 (12) | 1 (4) |
| Neurosurgery, n (%) |  |  |  |  |  |
| Evacuation of hematomas | 23 (43) | 13 (50) | 10 (37) | 14 (56) | 8 (32) |
| Evacuation of hematomas and decompressive craniectomy | 7 (13) | 4 (15) | 3 (11) | 5 (20) | 2 (8) |
| Decompressive craniectomy | 10 (19) | 6 (23) | 4 (15) | 7 (28) | 3 (12) |
| Revision of skull fracture | 4 (8) | 2 (8) | 3 (11) | 2 (8) | 3 (12) |
| Dura reconstruction | 6 (11) | 5 (19) | 1 (4) | 4 (16) | 1 (4) |
| Type of trauma, n (%) |  |  |  |  |  |
| Multi trauma | 23 (43) | 10 (38) | 13 (48) | 8 (32) | 13 (52) |
| Isolated head trauma | 30 (57) | 16 (62) | 14 (52) | 17 (68) | 12 (48) |
| Cause of trauma, n (%) |  |  |  |  |  |
| Road traffic accident | 28 (53) | 12 (46) | 16 (59) | 11 (44) | 15 (60) |
| Fall | 16 (30) | 8 (31) | 8 (30) | 9 (36) | 6 (24) |
| Miscellaneous | 8 (15) | 6 (23) | 2 (7) | 5 (20) | 3 (12) |
| Assault | 1 (2) | 0 (0) | 1 (4) | 0 (0) | 1 (4) |
| Marshall CT-Classification, n (%), Missing=1 |  |  |  |  |  |
| Diffuse injury I (Normal) | 0 (0) | 0 (0) | 0 (0) | 0 (0) | 0 (0) |
| Diffuse injury II (Lesions, present cisterns, midline shift 0-5mm) | 14 (28) | 6 (23) | 8 (30) | 6 (24) | 8 (32) |
| Diffuse injury III (Lesions, cisterns compressed, midline shift 0-5mm) | 11 (21) | 5 (19) | 6 (22) | 4 (16) | 6 (24) |
| Diffuse injury IV (Midline shift > 5 mm) | 24 (45) | 13 (50) | 11 (41) | 12 (48) | 10 (40) |
| V (Any surgically evacuated lesion) | 3 (6) | 2 (8) | 1 (4) | 3 (12) | 0 (0) |
| VI (Non evacuated mass lesion, >25cc lesion) | 0 (0) | 0 (0) | 0 (0) | 0 (0) | 0 (0) |
| Rehabilitation, n (%), Missing=2 | 37 (70) | 15 (58) | 22 (81) | 14 (56) | 22 (88) |
| Patient characteristics presented for the whole cohort (total) and in subgroups, GOS 1-3 and GOS 4-5 outcome at both one year and 10-15 years after trauma. At the assessment 10-15 years after trauma, 3 patients did not participate, i.e. 10-15 years after trauma: missing=3, n=50. Neurological diseases include epilepsy (n=5), transient ischemic attack (n=1) and previous trauma to the head (n=2). For categorical variables n (%) is presented. For continuous variables Mean (SD) / Median (Min; Max) is presented. GOS: Glasgow Outcome Scale, n: number of observations. | | | | | |

| Supplement 2: Table 2. Correlations between potential confounders, NFL and GFAP and outcome one year and 10-15 years after trauma | | | | | | | | | | | | |
| --- | --- | --- | --- | --- | --- | --- | --- | --- | --- | --- | --- | --- |
|  | GOS 1 year  after trauma | | | GOS 10-15 years  after trauma | | | NfL (μg/L) | | | GFAP (μg/L) | | |
|  | Spearman’s correlation coefficient | P-value | n | Spearman’s correlation coefficient | P-value | n | Spearman’s correlation coefficient | P-value | n | Spearman’s correlation coefficient | P-value | n |
| Age at time of trauma | -0.424 | 0.0015 | 53 | -0.443 | 0.0013 | 50 | 0.236 | 0.0882 | 53 | 0.429 | 0.0013 | 53 |
| Sex | 0.0131 | 0.926 | 53 | 0.100 | 0.488 | 50 | 0.299 | 0.0294 | 53 | 0.271 | 0.0496 | 53 |
| Neurological diseases | -0.122 | 0.393 | 51 | -0.315 | 0.0288 | 48 | 0.165 | 0.248 | 51 | 0.282 | 0.0449 | 51 |
| Diabetes mellitus | -0.00970 | 0.945 | 53 | -0.134 | 0.351 | 50 | 0.182 | 0.192 | 53 | 0.0607 | 0.666 | 53 |
| Heart diseases | -0.220 | 0.112 | 53 | -0.323 | 0.0221 | 50 | 0.0467 | 0.740 | 53 | 0.0467 | 0.740 | 53 |
| High blood pressure | -0.127 | 0.363 | 53 | -0.0615 | 0.671 | 50 | 0.123 | 0.381 | 53 | 0.133 | 0.341 | 53 |
| Marshall CT classification | -0.0590 | 0.678 | 52 | -0.0771 | 0.598 | 49 | 0.242 | 0.0835 | 52 | 0.190 | 0.177 | 52 |
| Rehabilitation | -0.484 | 0.0003 | 51 | -0.316 | 0.0268 | 49 | 0.275 | 0.0512 | 51 | 0.322 | 0.0210 | 51 |
| Type of injury | 0.0931 | 0.507 | 53 | 0.155 | 0.281 | 50 | -0.0796 | 0.571 | 53 | -0.0298 | 0.832 | 53 |
| Spearman’s correlation coefficient was calculated on the logarithmic value of NfL and GFAP, using the highest measured value of NfL and GFAP. To be classified as a confounder, a variable need to relate to NfL and/or GFAP and GOS one year and /or GOS 10-15 years after trauma. Rehabilitation is not a confounder but an effect modifier. Age, NfL and GFAP are continuous variables, Marshall CT classification (6-grade) and GOS (5-grade) are ordinal, all other variables are dichotomous (Yes/No) and type of injury (isolated head injury or multi trauma). Neurological diseases, diabetes mellitus, high blood pressure and heart diseases are pre-trauma. CSF: Cerebral Spinal Fluid, GFAP: Glial Fibrillary Acidic Protein, GOS: Glasgow Outcome Scale, n: number of observations, NfL: Neurofilament Light. | | | | | | | | | | | | |

| Supplement 2: Table 3.  Comparison of initial maximal NfL and GFAP concentrations between poor vs. good outcome and dead vs. alive one year and 10-15 years after trauma. | Adjusted | Fold change (95% CI) | One year after trauma | 0.20  (0.07-0.62) | | 0.10  (0.03-0.35) | | 10-15 years after trauma | 0.63  (0.23-1.69) | | 0.69  (0.22-2.21) | | Data are presented as the median (IQR) for numeric variables. *Fisher’s Non-Parametric Permutation test was used to compare concentrations of NfL and GFAP between poor vs good outcome and dead vs alive; otherwise ANCOVA was used. Adjusted for age one year after trauma and age and neurological diseases 10-15 years after trauma. Each patients maximum CSF concentration of NfL and GFAP was used in the calculation. Abbreviations: CI: confidence interval, CSF: Cerebral Spinal Fluid, GFAP: Glial Fibrillary Acidic Protein, GOS: Glasgow Outcome Scale, IQR: interquartile range n: number of observations, NfL: Neurofilament Light. |
| --- | --- | --- | --- | --- | --- | --- | --- | --- | --- | --- | --- | --- | --- |
|  |  | P-value |  | 0.006 | | <.001 | |  | 0.35 | | 0.53 | |  |
|  | Unadjusted | Fold change (95% CI) |  | 0.17  (0.06-0.46) | | 0.05  (0.02-0.19) | |  | 0.43  (0.18-0.98) | | 0.31  (0.10-0.93) | |  |
|  |  | P-value* |  | <.001 | | <.001 | |  | 0.047 | | 0.037 | |  |
|  |  | GOS 2–5 (Alive) |  | 6.21  (2.3–15.1) | n=44 | 59.9  (17.0–198) | n=44 |  | 6.09  (2.4–15.9) | n=33 | 61.9  (29.4–119) | n=33 |  |
|  |  | GOS 1 (Dead) |  | 44.4  (14.7–64.2) | n=9 | 2120  (545–3720) | n=9 |  | 13.1  (6.20–39.2) | n=20 | 240  (33.8–1620) | n=20 |  |
|  | Adjusted | Fold change (95% CI) |  | 0.33  (0.15-0.73) | | 0.29  (0.11-0.77) | |  | 0.38  (0.15-0.95) | | 0.41  (0.13-1.24) | |  |
|  |  | P-value |  | 0.007 | | 0.015 | |  | 0.038 | | 0.11 | |  |
|  | Unadjusted | Fold change (95% CI) |  | 0.28  (0.13-0.59) | | 0.18  (0.07-0.49) | |  | 0.30  (0.14-0.65) | | 0.21  (0.07-0.60) | |  |
|  |  | P-value* |  | 0.003 | | 0.002 | |  | 0.005 | | 0.004 | |  |
|  |  | GOS 4–5 (Good outcome)  outcome |  | 3.70  (1.50–14.1) | n=27 | 43.8  (10.4–89.5) | n=27 |  | 5.96  (2.40–14.1) | n=25 | 50.3  (18.0–86.5) | n=25 |  |
|  |  | GOS 1–3 (Poor)  outcome |  | 15.5  (6.30–34.6) | n=26 | 146  (83.6–1770) | n=26 |  | 20.6  (10.9–34.6) | n=25 | 222  (55.7–1770) | n=25 |  |
|  |  | Variable |  | NfL  (μg/L) | | GFAP (μg/L) | |  | NfL  (μg/L) | | GFAP (μg/L) | |  |

**Supplement 2: Fig 1. Changes in GOS to 10-15 years after trauma by GOS at one year.**


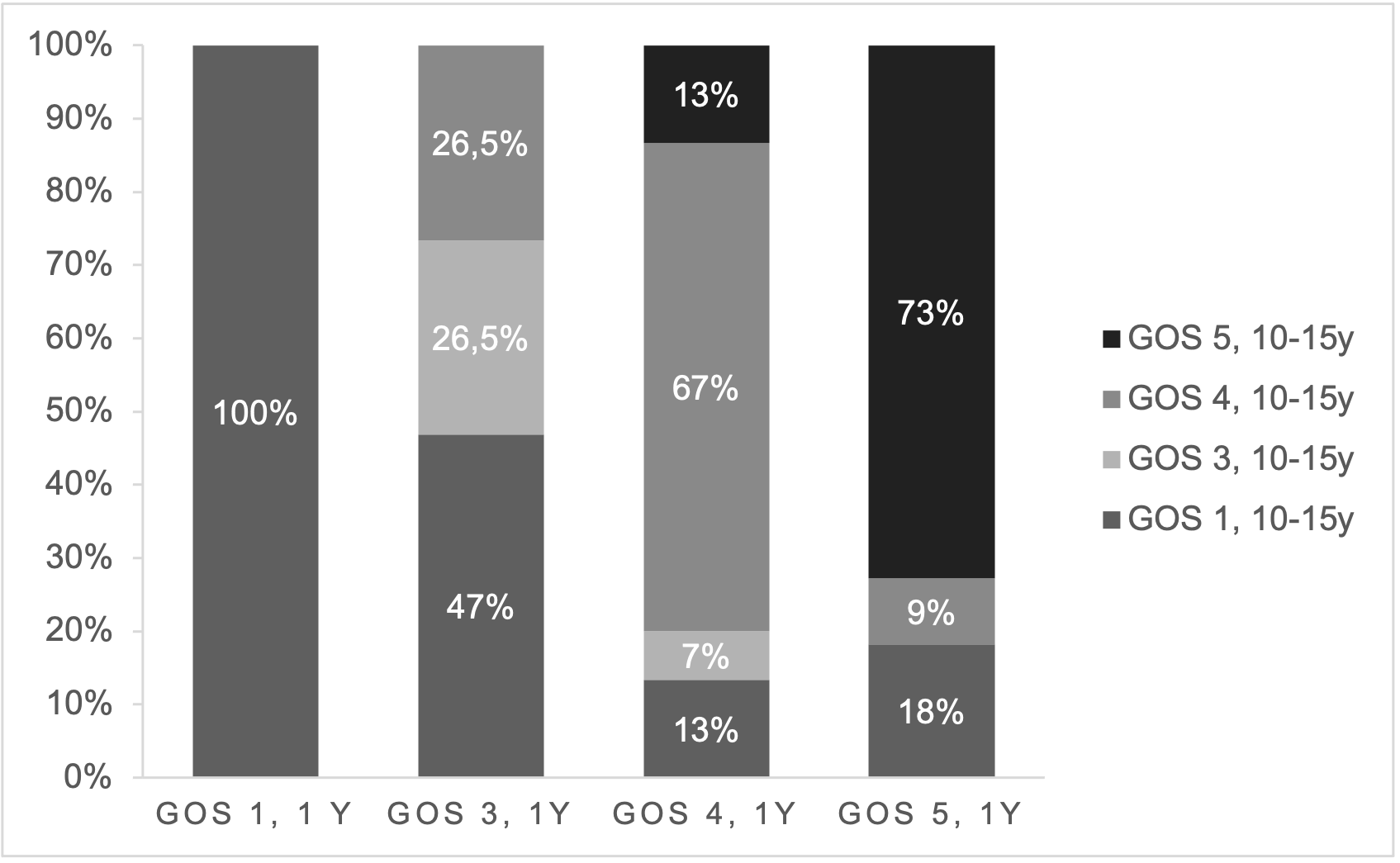


Each bar represents the GOS at one year. Within each bar, the distribution of GOS classifications at 10-15 years after trauma is shown (in percent). GOS: Glasgow Outcome Scale.

**Supplement 2: Fig 2. Trajectory profile of NfL and GFAP (μg/L) in CSF.**


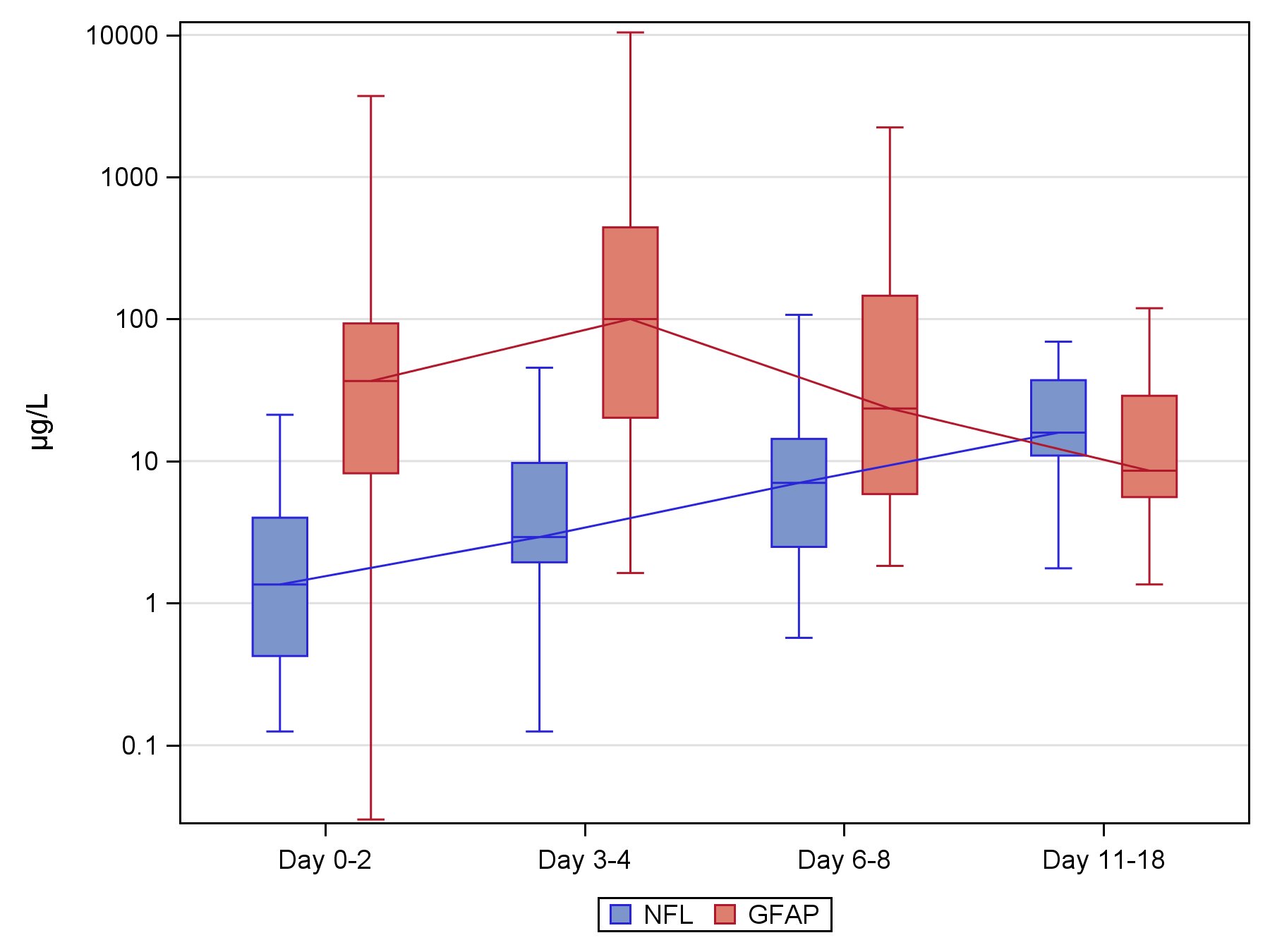


Concentrations of NfL and GFAP in the CSF are shown per sample period in boxplots. The median concentrations per sample period are connected to illustrate the trajectory profile of each biomarker. Concentrations of NfL and GFAP are presented in μg/L and on a log10 scale. The sample period is expressed in days after trauma. The number of samples were day 0-2 (n=24), day 3-4 (n=33), day 6-8 (n=35) and day 11-18 (n=15). CSF: Cerebral Spinal Fluid, GFAP: Glial Fibrillary Acidic Protein, NfL: Neurofilament Light.

**Supplement 2: Fig 3a, b. Concentrations of NfL and GFAP in each GOS category.**

**
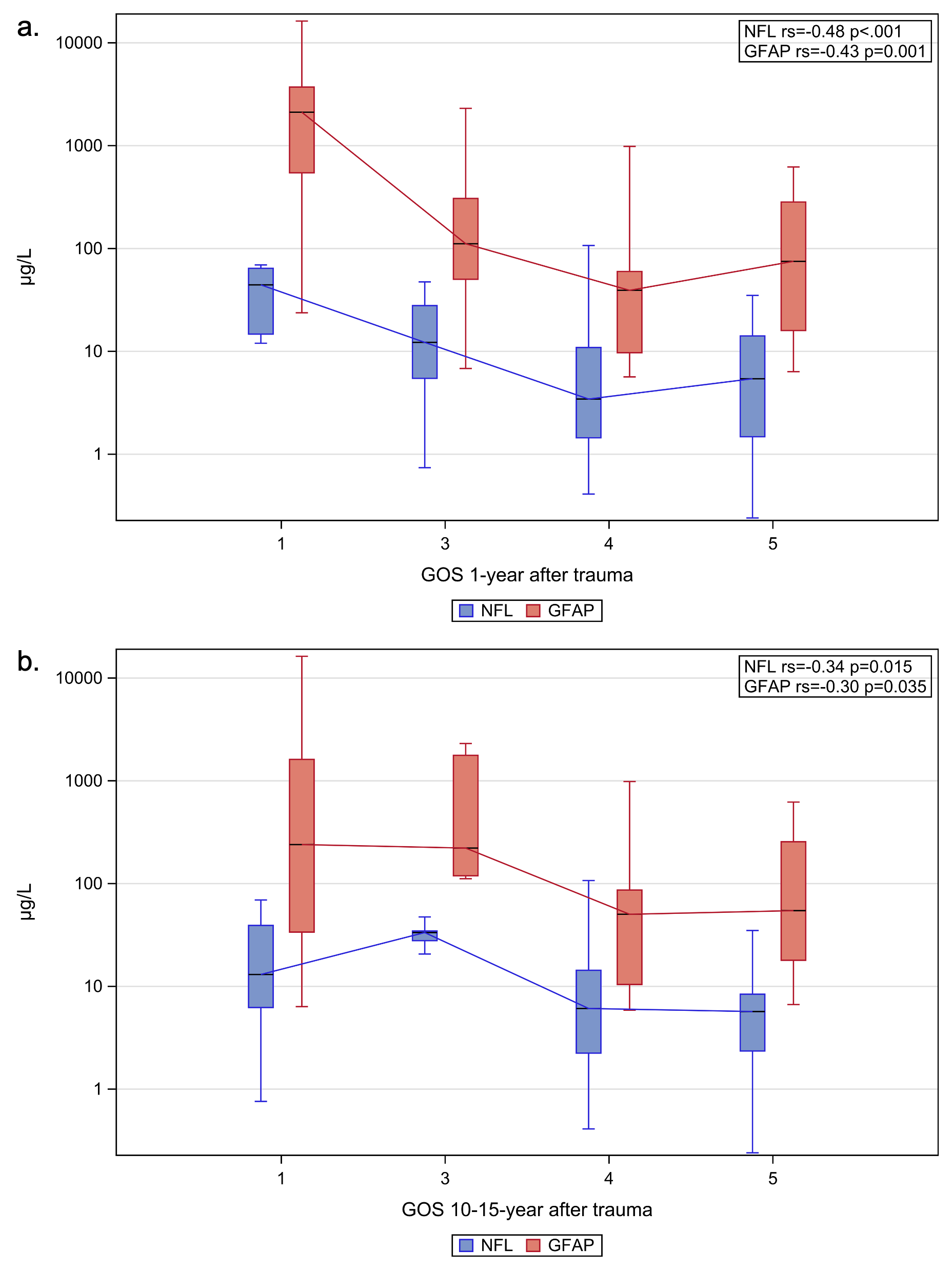
**

Boxplot presenting initial CSF concentrations of NfL and GFAP in each GOS category 1-5. a. GOS one year after trauma, b. GOS 10-15 years after trauma. Each patients maximum CSF concentration of NfL and GFAP was used in the calculation. CSF: Cerebral Spinal Fluid, GFAP: Glial Fibrillary Acidic Protein, GOS: Glasgow Outcome Scale, NfL: Neurofilament Light.

**Supplement 2: Fig 4a-d. Concentrations of NfL and GFAP in those with GOS 1-3 (poor) vs. those with GOS 4-5 (good outcome) and those with GOS 1 (dead) vs. those with GOS 2-5 (alive), one year and 10-15 years after trauma.**

**
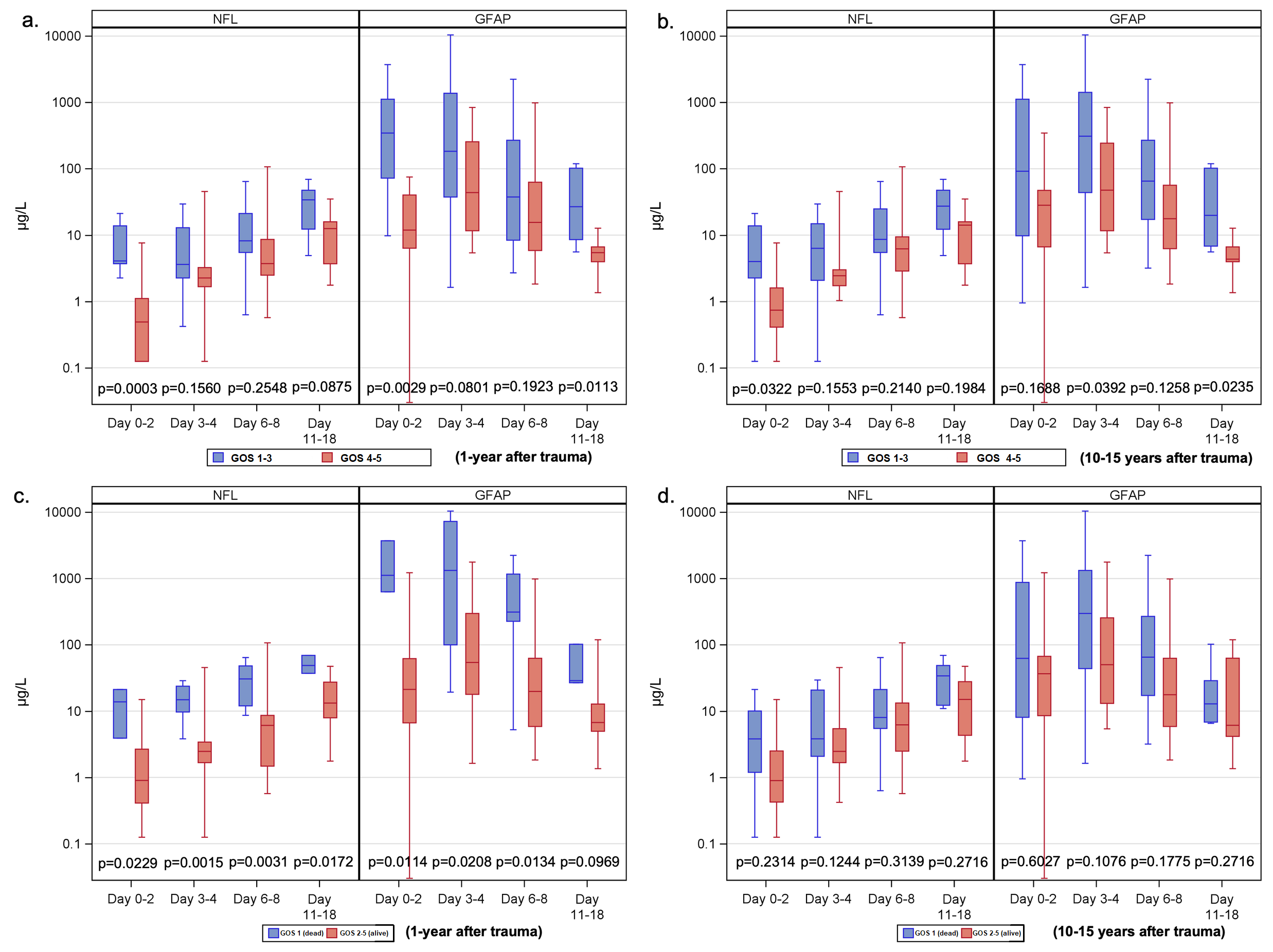
**

Boxplot presenting initial CSF concentrations of NfL and GFAP (μg/L on a log10 scale), separately, per sample period. Concentrations of NfL and GFAP in CSF, in stratified groups: GOS 1-3 vs. GOS 4-5 outcome a. One year after trauma b. 10-15 years after trauma and GOS 1 vs. GOS 2-5 c. One year after trauma d. 10-15 years after trauma. Each patients maximum CSF concentration of NfL and GFAP was used in the calculation. CSF: Cerebral Spinal Fluid, GFAP: Glial Fibrillary Acidic Protein, GOS: Glasgow Outcome Scale, NfL: Neurofilament Light.

**Supplement 2: Fig 5a-d. The OR for GOS 1-3 (poor) outcome and GOS 1 (dead) one year and 10-15 years after trauma by NfL and GFAP.**


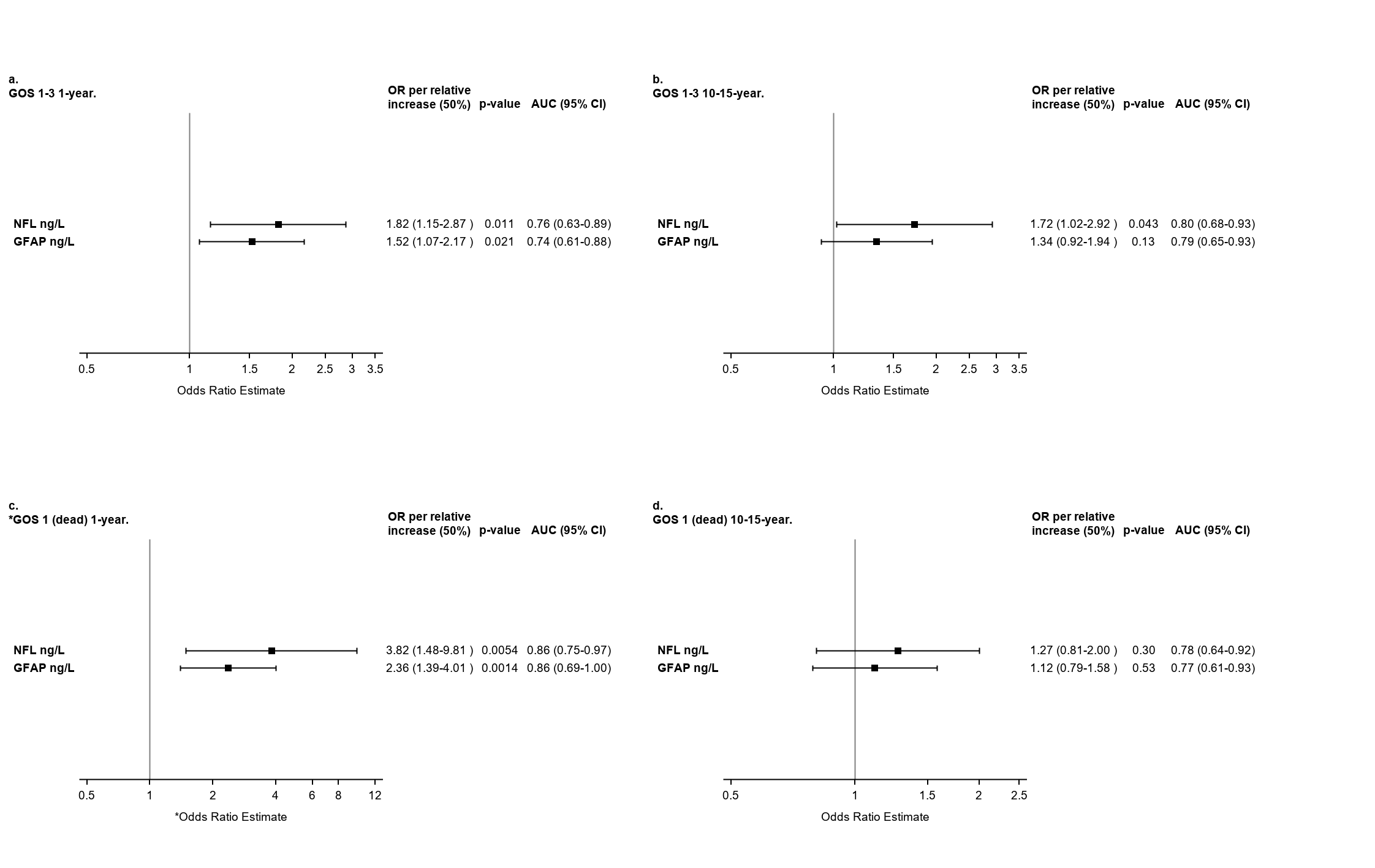


The OR is the ratio of the odds for the dependent variable of GOS 1-3 outcome or GOS 1 with a relative increase in initial NfL or GFAP in the CSF of 50%. a. GOS 1-3 outcome one year after trauma b. GOS 1-3 outcome 10-15 years after trauma c. GOS 1 one year after trauma and d. GOS 1 10-15 years after trauma. *The analysis was unadjusted due to few events. GOS 1-3 outcome at one year is adjusted for age. GOS 1-3 outcome and GOS 1 10-15 years after trauma are adjusted for age and neurological diseases. The results for OR, p-value and area under the ROC curve (AUC) are based on original values and not stratified groups. Each patients maximum CSF concentration of NfL and GFAP was used in the calculation. CSF: Cerebral Spinal Fluid, GFAP: Glial Fibrillary Acidic Protein, GOS: Glasgow Outcome Scale, NfL: Neurofilament Light.

**Supplement 2: Fig 6 a-d. Receiver operating curve (ROC) for NfL and GFAP, in the CSF, for GOS 1-3 outcome and GOS 1 outcome.**

**
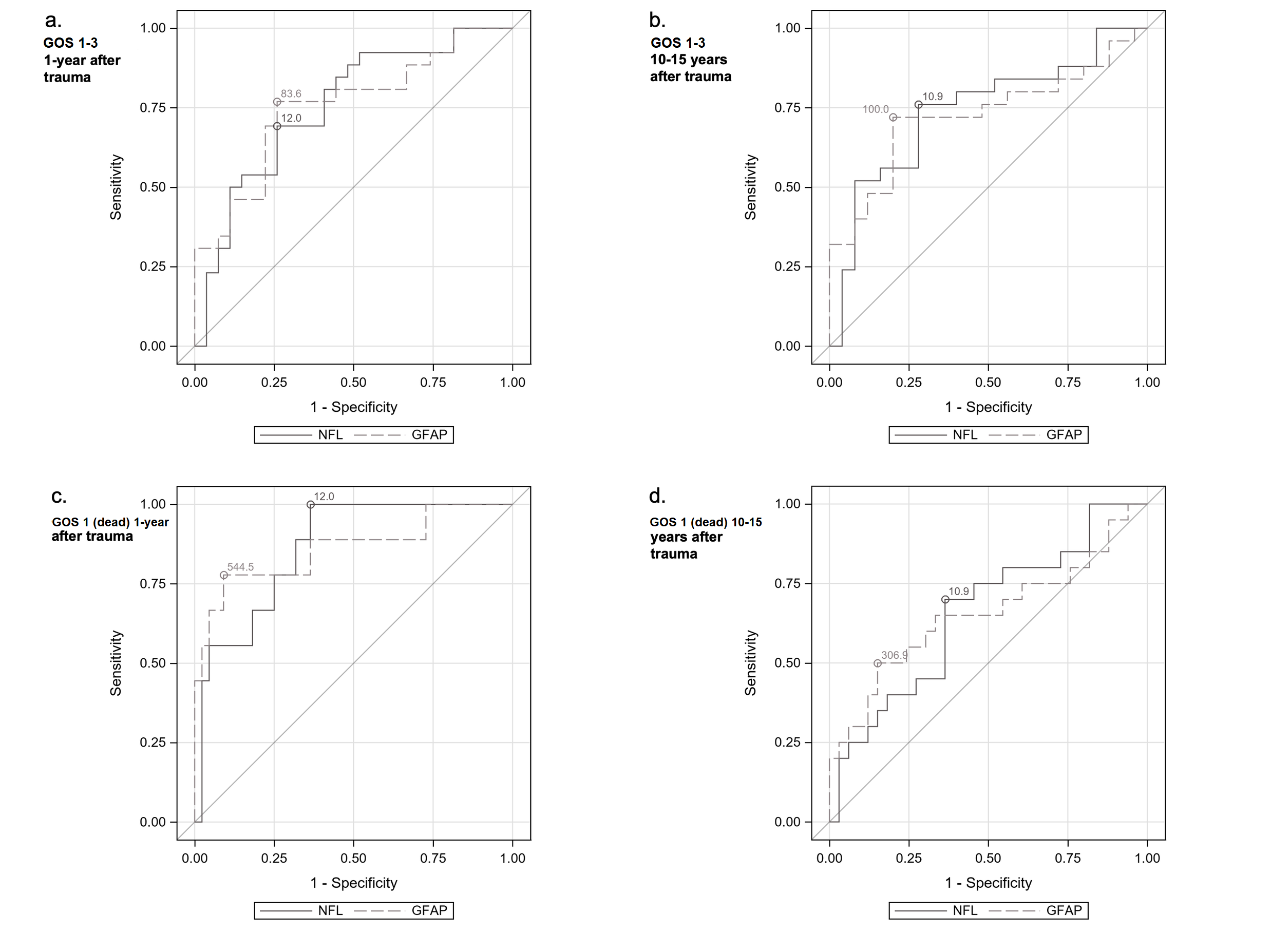
**

Receiver operating curve illustrating the quality at which specific levels of NfL and GFAP in the CSF can separate GOS 1-3 from GOS 4-5 outcome and GOS 1 outcome from GOS 2-5 outcome. a. GOS 1 outcome one year after trauma b. GOS 1-3 outcome 10-15 years after trauma c. GOS 1 one year after trauma and d. GOS 1 10-15 years after trauma. The marked cut-off value is calculated using the sum of best sensitivity and specificity for each biomarker. Each patients maximum CSF concentration of NfL and GFAP was used in the calculation. CSF: Cerebral Spinal Fluid, GFAP: Glial Fibrillary Acidic Protein, GOS: Glasgow Outcome Scale, NfL: Neurofilament Light.
